# Propagative α-synuclein seeds as serum biomarkers for synucleinopathies

**DOI:** 10.1038/s41591-023-02358-9

**Published:** 2023-05-29

**Authors:** Ayami Okuzumi, Taku Hatano, Gen Matsumoto, Shuko Nojiri, Shin-ichi Ueno, Yoko Imamichi-Tatano, Haruka Kimura, Soichiro Kakuta, Akihide Kondo, Takeshi Fukuhara, Yuanzhe Li, Manabu Funayama, Shinji Saiki, Daisuke Taniguchi, Taiji Tsunemi, Deborah McIntyre, Jean-Jacques Gérardy, Michel Mittelbronn, Rejko Kruger, Yasuo Uchiyama, Nobuyuki Nukina, Nobutaka Hattori

**Affiliations:** 1 Department of Neurology, Juntendo University Faculty of Medicine, Tokyo, Japan; 2 Department of Histology and Cell Biology, Nagasaki University School of Medicine, Nagasaki, Japan; 3 Medical Technology Innovation Center, Juntendo University Faculty of Medicine, Tokyo, Japan; 4 Laboratory of Morphology and Image Analysis, Biomedical Research Core Facilities, Juntendo University Faculty of Medicine, Tokyo, Japan; 5 Department of Neurosurgery, Juntendo University Faculty of Medicine, Tokyo, Japan; 6 Neurodegenerative Disorders Collaboration Laboratory, RIKEN Center for Brain Science, Saitama, Japan; 7 Department of Neurology, Institute of Medicine, University of Tsukuba, Tsukuba, Japan; 8 Transversal Translational Medicine, Luxembourg Institute of Health (LIH), Strassen, Luxembourg; 9 Luxembourg National Center of Pathology (NCP), Laboratoire National de Santé (LNS); Department of Cancer Research (DOCR), Luxembourg Institute of Health (LIH); Luxembourg Centre of Neuropathology (LCNP), Luxembourg Centre for Systems Biomedicine (LCSB), Faculty of Science, Technology and Medicine (FSTM) and Department of Life Sciences and Medicine (DLSM), University of Luxembourg, Esch-sur-Alzette, Luxembourg; 10 Centre Hospitalier de Luxembourg (CHL); Translational Neuroscience, Luxembourg Centre for Systems Biomedicine (LCSB), University of Luxembourg, Strassen, Luxembourg; 11 Department of Cellular and Molecular Neuropathology, Juntendo University Faculty of Medicine, Tokyo, Japan; 12 Laboratory of Structural Neuropathology, Graduate School of Brain Science, Doshisha University, Kyoto, Japan

**Keywords:** Parkinson's disease, Cellular neuroscience

## Abstract

Abnormal α-synuclein aggregation is a key pathological feature of a group of neurodegenerative diseases known as synucleinopathies, which include Parkinson’s disease (PD), dementia with Lewy bodies and multiple system atrophy (MSA). The pathogenic β-sheet seed conformation of α-synuclein is found in various tissues, suggesting potential as a biomarker, but few studies have been able to reliably detect these seeds in serum samples. In this study, we developed a modified assay system, called immunoprecipitation-based real-time quaking-induced conversion (IP/RT-QuIC), which enables the detection of pathogenic α-synuclein seeds in the serum of individuals with synucleinopathies. In our internal first and second cohorts, IP/RT-QuIC showed high diagnostic performance for differentiating PD versus controls (area under the curve (AUC): 0.96 (95% confidence interval (CI) 0.95–0.99)/AUC: 0.93 (95% CI 0.84–1.00)) and MSA versus controls (AUC: 0.64 (95% CI 0.49–0.79)/AUC: 0.73 (95% CI 0.49–0.98)). IP/RT-QuIC also showed high diagnostic performance in differentiating individuals with PD (AUC: 0.86 (95% CI 0.74–0.99)) and MSA (AUC: 0.80 (95% CI 0.65–0.97)) from controls in a blinded external cohort. Notably, amplified seeds maintained disease-specific properties, allowing the differentiation of samples from individuals with PD versus MSA. In summary, here we present a novel platform that may allow the detection of individuals with synucleinopathies using serum samples.

## Abstract (web summary)

A modified seed aggregation assay detects minute amounts of serum α-synuclein seeds in individuals with synucleinopathy, demonstrating high performance as a diagnostic biomarker.

## Main

Synucleinopathies, including Parkinson’s disease (PD), dementia with Lewy bodies (DLB) and multiple system atrophy (MSA), are neurodegenerative disorders characterized by the abnormal aggregation of α-synuclein^1–4^. Additionally, rapid eye movement sleep behavior disorder (RBD) is a prodromal symptom of synucleinopathy^5^. In the pathologies of synucleinopathy, α-synuclein misfolding and aggregation involve a mechanism of seeding, in which initial seeds of α-synuclein, known as fibrils, recruit soluble monomers that form visible aggregates^2,6^ such as Lewy bodies (LB), a histopathological hallmark of PD, or neuronal cytoplasmic inclusions and oligodendroglial cytoplasmic inclusions in MSA^7,8^. Furthermore, abnormal α-synuclein aggregations are observed in systemic autonomic neurons in patients with synucleinopathy. Thus, it is possible that α-synuclein fibrils exist in the intricate systemic network, including the nervous, lymphatic and vascular systems^9^. Several studies revealed increased α-synuclein in the sera of patients with PD compared to healthy controls by ELISA, immunomagnetic reduction or electrochemiluminescence immunoassay^10–13^. Aptamer DNA-PAINT combined with single-aggregate confocal fluorescence methods revealed that some of the serum α-synucleins have abnormal β-sheet structures^14^. These findings suggest that α-synuclein fibrils in the serum of patients with synucleinopathies might be present in the blood, although these assays have not been available as diagnostic biomarkers of synucleinopathies.

Synucleinopathy-specific α-synuclein fibrils were detected in cerebrospinal fluid (CSF)^1,15–20^, plasma neuron-derived exosomes^21^, saliva gland^22^, skin^23,24^ and olfactory mucosa^25–27^ by the amplification of abnormal α-synuclein aggregation using real-time quaking-induced conversion (RT-QuIC) assays, which takes advantage of the seeding properties to amplify small quantities of seeds. In this study, we developed a modified RT-QuIC assay combined with immunoprecipitation (IP) to concentrate α-synuclein seeds from serum: the IP-based RT-QuIC (IP/RT-QuIC) assay.

We validated the IP/RT-QuIC usefulness as a diagnostic marker of synucleinopathies and propose here that the fibril morphology of products derived from IP/RT-QuIC of serum α-synuclein seeds in patients with synucleinopathies could discriminate among PD, DLB and MSA. We detected the serum α-synuclein seeds by IP/RT-QuIC in synucleinopathies and found the existence of the different conformational strains in the serum of patients with PD, DLB and MSA.

## Results

### Participants

The sera from 270 patients with synucleinopathy (221 PD, 39 MSA and 10 DLB), 9 patients with RBD (longitudinal IP/RT-QuIC was conducted for 3 of them), 128 non-neurodegenerative controls, 30 patients with progressive supranuclear palsy (PSP), 25 patients with Alzheimer’s disease (AD) and 17 patients with Parkin-linked PD (*PRKN*) were analyzed. The second internal cohort, which was recruited in our previous metabolomics investigation^28^, included 40 patients with synucleinopathy (34 PD and 6 MSA) and 9 non-neurodegenerative controls. We also analyzed an external cohort including a blinded analysis of 35 participants with synucleinopathies (20 PD and 15 MSA), 20 non-neurodegenerative controls and six patients with tauopathy as non-synucleinopathy controls. We additionally analyzed sera collected from internal and external cohorts of seven patients with pathologically confirmed synucleinopathies (three PD with dementia (PDD), three MSA from the internal cohort and one PDD from the external cohort), two pathologically verified patients with tauopathies as non-synucleinopathy controls (external cohort) and three age-matched controls (internal cohort). These patients’ clinical data are shown in Table 1 and Extended Data Table 1. In this study, sex and/or gender data were determined and assigned based on medical records.

**Table 1:** Characteristics of the study participants

	Age (years), mean (s.d.)	Men, *n* (%)	Hoehn–Yahr stage, mean (s.d.)	UPDRS-III, mean (s.d.)	Disease duration (years), mean (s.d.)
PD (*n* = 221)	66 (10)	98 (44)	2.1 (1.0)	13 (10)	6.9 (5.8)
MSA (*n* = 39)	64 (8.5)	15 (38)	2.9 (1.3)	35 (20)	3.2 (2.3)
DLB (*n* = 10)	76 (5.3)	6 (60)	2.1 (0.6)	19 (9.4)	5.4 (3.6)
RBD (*n* = 9)	73 (6.2)	4 (44)	NA	3 (7.5)	8.8 (7.4)
PSP (*n* = 30)	72 (9.7)	13 (43)	3.5 (1.1)	44 (21)	4.7 (2.5)
AD (*n* = 25)	75 (9.0)	10 (40)	NA	NA	NA
PRKN (*n* = 17)	52 (18)	6 (35)	2.3 (0.7)	20 (21)	23 (12)
Controls (*n* = 128)	64 (15)	61 (47)	NA	NA	NA

NA, not applicable.

**Table 2:** Serum α-synuclein IP/RT-QuIC results and α-synuclein IP/RT-QuIC assay characteristics per diagnosis

Diagnosis	*n*	IP/RT-QuIC results +/−	Positive results
Synucleinopathies			
PD	221	210/11	95%
MSA	39	25/14	64%
DLB	10	9/1	90%
RBD	9	4/5	44%
Non-synucleinopathies			
PSP	30	1/29	3%
AD	25	4/21	16%
PRKN	17	0/17	0%
Controls	128	11/117	8.5%

Data are presented as numbers. *n*, number of participants who received IP/RT-QuIC.

### Detection of α-synuclein seeds in serum

We examined the sensitivity to detect small amounts of α-synuclein seeds with parameters of IP/RT-QuIC assay. The time to threshold (*T*_1/2_ and *T*_max_) or area under the curve (AUC) depended on the initial α-synuclein seed concentration. We revealed that IP/RT-QuIC would detect a concentration of 1,000 pg ml^−1^ or higher. However, the substrate lot was critical because the unsuitable α-synuclein recombinant protein might be self-aggregated (false positive) or fail to convert to seed (false negative). The repetitive examinations concluded the detection limit as 1,000 pg ml^−1^. We defined the forming rate as the rate of aggregate formation that was determined as a slope of the tangent line at the inflection point of the sigmoid curve of the IP/RT-QuIC. The forming rates did not depend on the concentration (Supplementary Fig. 1).

**Fig. 1:**
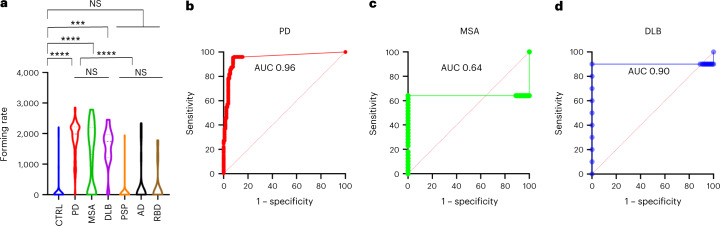
Parameters describing the kinetics of α-synuclein aggregation in the serum IP/RT-QuIC and ROC analysis of the diagnostic performance of serum IP/RT-QuIC for synucleinopathy. **a**, Comparison of the forming rates of each group. The violin plots match those represented in the kinetic curves. Violin plots show the range and average distribution. The symbols indicate outliers according to Tukey’s test. Statistical analysis was performed using two-sided one-way ANOVA with Tukeyʼs multiple comparisons test, resulting in a significance of *P* < 0.001 (***) and *P* < 0.0001 (****) between each group (CTRL versus PD, *P* < 0.0001; CTRL versus MSA, *P* < 0.0001; CTRL versus DLB, *P* = 0.0001; CTRL versus PSP, *P* = 0.9914; CTRL versus AD, *P* = 0.8547; CTRL versus RBD, *P* = 0.9300; PD versus MSA, *P* = 0.9971; PD versus DLB, *P* = 0.9277; MSA versus DLB, *P* = 0.8415; PSP versus AD, *P* = 0.6755; PSP versus RBD, *P* = 0.9660; AD versus RBD, *P* > 0.9999; PD, MSA and DLB versus PSP, AD and RBD, *P* < 0.0001). **b–d**, ROC curves for serum IP/RT-QuIC comparing the control group to PD (**b**), MSA (**c**) and DLB (**d**). NS, not significant.

**Extended Data Fig. 1:**
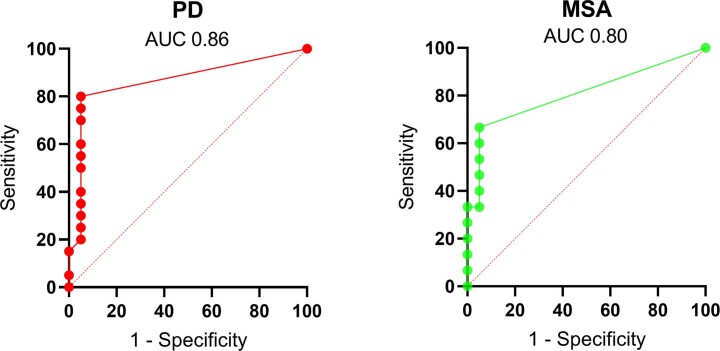
ROC analysis of the diagnostic performance of serum IP/RT-QuIC for synucleinopathy in the external cohort. ROC curves for serum IP/RT-QuIC comparing the control group with PD and MSA groups. AUC, the area under the curve; MSA, multiple system atrophy; PD, Parkinson’s disease.

Next, we investigated the serum using IP/RT-QuIC (designated as serum IP/RT-QuIC) and identified α-synuclein seeds in synucleinopathy (Table 2 and Extended Data Table 4). Positive results for serum IP/RT-QuIC for patients with PD in the first, second and external cohorts were 210/221 (95%), 34/34 (100%) and 15/20 (75%), respectively (Table 2 and Extended Data Table 4). Negative results were found in 31 of 279 patients with synucleinopathy in the first cohort (11/221 PD, 14/39 MSA, 1/10 DLB and 5/9 RBD; Table 2 and Extended Data Table 3); 2 of 40 patients in the second internal cohort (0/34 PD and 2/6 MSA); and 12 of 35 patients in the external cohort (5/20 PD and 7/15 MSA) (Extended Data Table 4a,b). Results of the assay reproducibility analysis are provided in the Supplementary Methods (Supplementary Tables 1–5). Seventeen of 26 patients with MSA parkinsonian variant (MSA-P) and eight of 13 patients with MSA cerebellar variant (MSA-C) were positive (Supplementary Table 6). Positive results were found in five of 55 non-synucleinopathy patients (1/30 PSP and 4/25 AD) and 11 of 128 controls. The average age of the 11 control cases with positive IP/RT-QuIC results was 75 years (50 years: one; 61–75 years: three; over 76 years: seven) (Supplementary Table 7). All *PRKN*-positive patients had negative IP/RT-QuIC results. The forming rates of IP/RT-QuIC results were significantly higher in patients with synucleinopathy than in controls or non-synucleinopathy patients (*P* < 0.001), but the comparison among patients with PD, DLB and MSA did not reveal significant results (Fig. 1a). In the receiver operating characteristic (ROC) analyses, the sensitivity and specificity for differentiating patients with PD from controls were 94.6% and 92.1%, respectively (cutoff: forming rate 662.4, AUC: 0.96 (95% confidence interval (CI) 0.95–0.99)). The sensitivity and specificity for differentiating patients with DLB from controls were 96.4% and 92.2%, respectively (cutoff: forming rate 574, AUC: 0.90 (95% CI 0.95–0.99)), and the sensitivity and specificity for differentiating patients with MSA from controls were 64.1% and 11.0%, respectively (cutoff: forming rate 118.4, AUC: 0.64 (95% CI 0.49–0.79)) (Fig. 1b–d). IP/RT-QuIC also had high diagnostic performance in the external cohort for differentiating patients with PD (cutoff: forming rate 650.2, AUC: 0.86 (95% CI 0.74–0.99)) and MSA (cutoff: forming rate 361, AUC: 0.80 (95% CI 0.65–0.97)) from controls (Extended Data Fig. 1). In the pathologically confirmed cases, positivity rates of IP/RT-QuIC for the cases with non-synucleinopathy, MSA and PD patients were 0/0 (0%), 1/3 (33%) and 3/3 (100%) in the internal cohort, respectively. In the external cohort, two non-synucleinopathy patients had negative results, whereas the patient with PDD had positive results (Extended Data Table 2). The correlation between IP/RT-QuIC parameters and clinical data in patients with PD (Extended Data Table 5a) and MSA (Extended Data Table 5b) was analyzed. The forming rates correlated with Unified Parkinson’s Disease Rating Scale Part III (UPDRS-III) (*P* = 0.028) and disease duration (*P* = 0.0051) in PD. There was no correlation between the other IP/RT-QuIC parameters and the clinical data. The results of Pearson’s chi-square tests showed a significant positive correlation between the results of the IP/RT-QuIC and delayed heart-to-mediastinum (H/M) ratio with ^123^I-metaiodobenzylguanidine (MIBG) cardiac scintigraphy in patients with PD (Supplementary Table 8a) but not in patients with MSA (Supplementary Table 8b). The rate of negative results of IP/RT-QuIC in patients with MSA was significantly higher than that in patients with PD (Table 2 and Supplementary Table 9).

### Morphological analysis of IP/RT-QuIC products

**Fig. 2:**
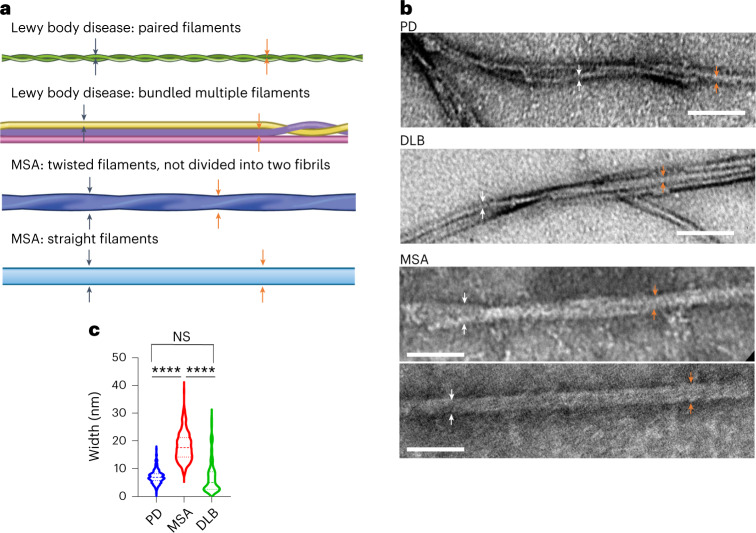
Structural differences between products of serum IP/RT-QuIC derived from participants with LB diseases or MSA. **a**, Schematic view of the main morphology of serum IP/RT-QuIC products derived from patients with LB diseases and MSA. Arrows indicate the measurement sites. **b**, Negative-stained TEM images of serum IP/RT-QuIC products derived from patients with PD, DLB and MSA. **c**, Violin plots show the range and average distribution. Statistical analysis was performed using one-way ANOVA followed by Tukey’s correction, resulting in significances of *P* < 0.0001 (****) among PD, DLB and MSA (PD versus MSA, *P* < 0.0001; PD versus DLB, *P* = 0.9514; MSA versus DLB, *P* < 0.0001). We measured the widths of 10 fibrils and took measurements at two sites for each fibril (PD (*n* = 50), MSA (*n* = 25), DLB (*n* = 9)). Scale bars, 100 nm. NS, not significant.

To investigate the morphological features of fibrils amplified by IP/RT-QuIC from the serum seeds of patients with LB disease (PD seeds and DLB seeds), MSA (MSA seeds) or RBD (RBD seeds), we performed a transmission electron microscopy (TEM) analysis using fibrils derived from randomly selected patients with IP/RT-QuIC-positive PD (*n* = 50), MSA (*n* = 25), DLB (*n* = 9) and RBD (*n* = 4) (Fig. 2 and Supplementary Fig. 2). The primary morphologies derived from PD seeds and DLB seeds were paired filaments or bundled multiple filaments (Fig. 2a,b), whereas, in MSA seeds, there were two distinct polymorphs: twisted filaments, which could not be divided into two filaments, and straight filaments (MSA-type filaments) (Fig. 2a,b). The fibril widths were different between the LB diseases (PD and DLB) and MSA (*P* < 0.0001) (Fig. 2c). All amplified fibrils derived from patients with RBD with positive IP/RT-QuIC results had the LB diseases-type structure (Supplementary Fig. 2). The single filaments of LB diseases and RBD seeds had narrower widths than those of MSA seeds (Fig. 2 and Supplementary Fig. 2). The cutoff value of widths was 11.86 nm. Under TEM, the α-synuclein fibrils from 11 IP/RT-QuIC-positive controls showed LB disease-type filaments with widths shorter than 11.86 nm (Supplementary Fig. 3). There were no significant differences in widths between α-synuclein fibrils of MSA-P and MSA-C (Supplementary Fig. 4).

**Extended Data Fig. 2:**
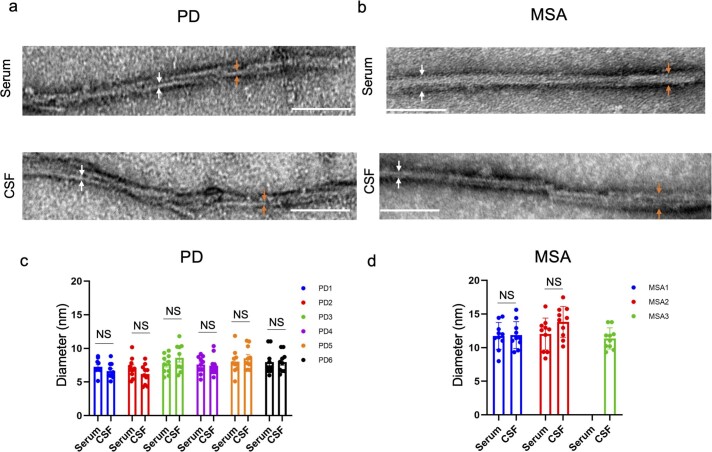
Morphological analysis of amplified products derived from the serum and CSF of PD and MSA cases. (a-d) Negative-stained transmission electron microscopy images of α-synuclein fibrils derived from serum and CSF by IP/RT-QuIC and RT-QuIC, respectively. **(a)** PD (case 2) and **(b)** MSA (case 2). The arrows indicate the measurement sites of the width. Scale bars are 100 nm. The widths of the amplified α-synuclein fibrils are shown for all **(c)** PD (n = 6: case 1, p = 0.5590; case 2, p = 0.7981; case 3, p = 0.7238; case 4, p = 0.7915 ; case 5, p = 0.3705; case 6, p = 0.6793) and **(d)** MSA cases (n = 3: case 1, p = 0.5801; case 2, p = 0.6237). The data represent mean ± SEM. Statistical analysis was conducted using a two-tailed t-test. We measured the width of 10 fibrils at two sites for each fibril. CSF, cerebrospinal fluid; MSA, multiple system atrophy; NS, not significant; PD, Parkinson’s disease.

To determine whether α-synuclein fibrils amplified from serum or CSF of patients with PD or MSA have any structural similarities, we compared the morphology of IP/RT-QuIC-amplified fibrils by TEM and confirmed that there are no structural differences between serum-derived and CSF-derived fibrils (Extended Data Fig. 2, Extended Data Table 6 and Supplementary Table 10).

### Structural analysis of seed-derived cellular inclusion

**Fig. 3:**
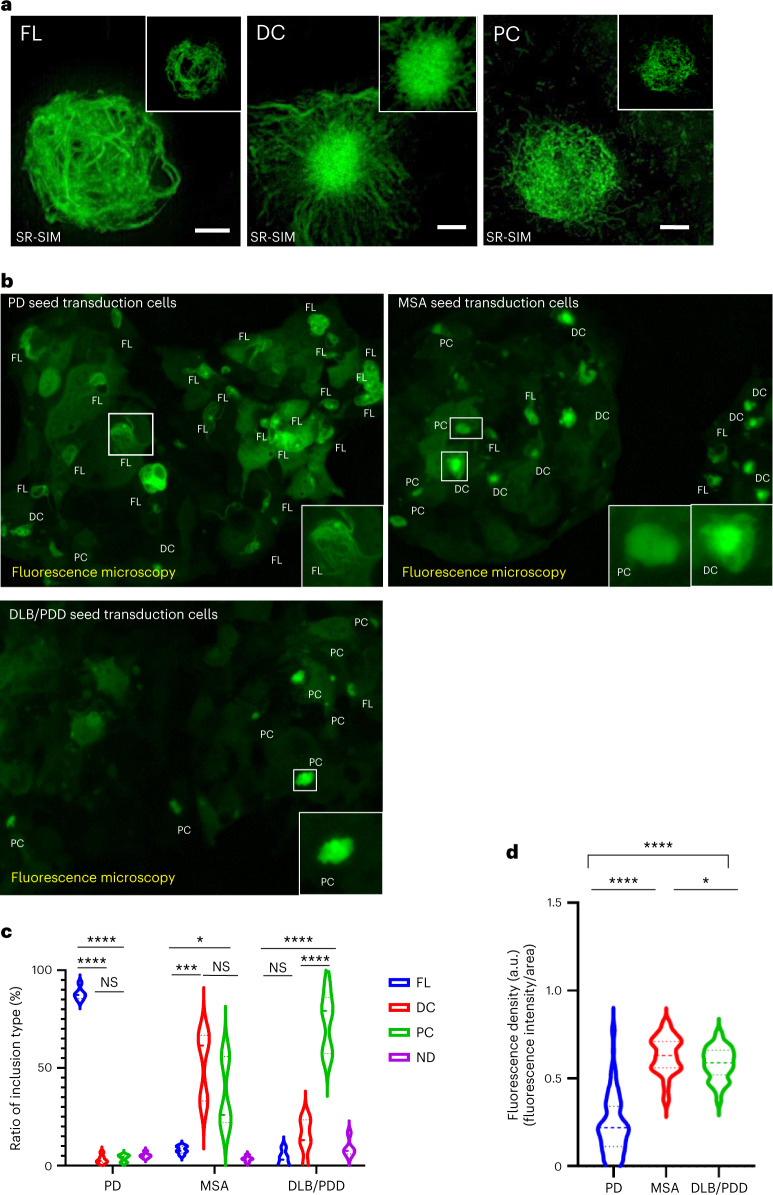
Serum IP/RT-QuIC-amplified α-synuclein seeds from participants with PD, MSA and DLB/PDD induce intracellular inclusions with distinct morphologies. **a**, Intracellular α-synuclein inclusions generated by serum IP/RT-QuIC products derived from patients with PD, MSA and DLB are visualized using SR-SIM. Representative morphologies of GFP-fused α-synuclein A53T inclusions generated by each disease-derived seed transduction in 293-SynG cells are shown. There are three different morphologies, including fibrous (FL), dense-core (DC) and pale-core (PC) inclusion (*n* = 5 different samples). The inset shows a single layer of the center slice of the inclusions. Scale bars are 2 μm, and the inset sides of the square are 10 µm. **b**, Images using BZ-X810 are generated as the full-focus image based on 10–20 *z*-stack images with 3-µm steps with a ×40 objective lens, and insets represent magnified images of inclusions in the square. Representative low-resolution images of 293-SynG cells transduced with each disease-derived seed. **c**, Violin plots indicate the ratio of each type of inclusion in five (MSA and DLB/PDD) or six (PD) independent low-resolution images obtained from each disease-specific seed transduced cell and evaluated in a blinded manner by three independent examiners as indicated. Statistical analysis was performed using a two-sided one-way ANOVA followed by Tukey’s correction, resulting in significances of *P* < 0.05 (*), *P* < 0.001 (***) and *P* < 0.0001 (****) among FL, DC, PC and ND among each synucleinopathy (PD: FL versus DC, *P* < 0.0001; FL versus PC, *P* < 0.0001; DC versus PC, *P* = 0.9596; MSA: FL versus DC, *P* = 0.0002; FL versus PC, *P* = 0.0131; DC versus PC, *P* = 0.2351; DLB/PDD: FL versus DC, *P* = 0.3194; FL versus PC, *P* < 0.0001; DC versus PC, *P* < 0.0001). **d**, The fluorescence density of intracellular α-synuclein inclusions, which was calculated as low-resolution fluorescence intensity of inclusion bodies divided by the area of inclusions, generated by seeds derived from patients with PD, MSA and DLB/PDD (PD versus MSA, *P* < 0.0001; PD versus DLB/PDD, *P* < 0.0001; MSA versus DLB/PDD, *P* = 0.0254) (*n* > 20 for each group). Violin plots show the range and average distribution. Statistical analysis was performed using a two-sided one-way ANOVA followed by Tukey’s correction, resulting in significances of *P* < 0.0001 (****) and *P* < 0.05 (*) among PD, MSA and DLB/PDD. ND, not determined; NS, not significant.

To confirm the seeding property of the amplified α-synuclein fibrils by serum IP/RT-QuIC, the α-synuclein fibrils were transduced into the HEK293 cell line stably expressing green fluorescent protein (GFP)-fused human α-synuclein with A53T mutation (293-SynG). As the 293-SynG cells never form intracellular inclusions without α-synuclein fibril transduction, the intracellular inclusion formation by the fibril transduction represents the seeding activity. We confirmed that all amplified α-synuclein fibrils by serum IP/RT-QuIC maintained the seeding property. Intriguingly, a super-resolution microscopy analysis revealed that intracellular inclusions created by the PD seeds, MSA seeds or DLB seeds tend to exhibit different morphological features, such as fibrous inclusions, dense-core inclusions in MSA and pale-core inclusions in DLB/PDD (Fig. 3a). PD seeds formed significantly more fibrous inclusions (*P* < 0.0001), and DLB/PDD seeds formed significantly more pale inclusions (*P* < 0.0001) (Fig. 3b,c). However, the morphological features of the inclusions were difficult to distinguish between dense-core and pale-core inclusions in low-magnification images. Conversely, filamentous inclusions were discriminated clearly from other inclusion morphologies (Fig. 3b,c). To objectively evaluate these inclusions, we measured the fluorescence density of the intracellular inclusion bodies, which was calculated by fluorescence intensity divided by the area of the inclusion bodies (Supplementary Fig. 5). This analysis confirmed that the fluorescence density of PD inclusions was significantly lower than that of DLB and MSA inclusions (Fig. 3d). To confirm whether the classified inclusion types correlated with disease pathology, three examiners performed the morphological assessment in a blinded manner in pathologically confirmed patients. The kappa coefficient of inter-batch analysis confirmed the reproducibility of the assessment (Supplementary Table 11), revealing that the amplification of α-synuclein fibrils from the patients’ serum samples and morphological typing of intracellular inclusions would be reliable (Fig. 3 and Supplementary Table 11) and that the cell-based assay might be a useful method for differentiating PD from MSA and DLB.

### Propagation of the amplified α-synuclein fibrils in vivo

**Extended Data Fig. 3:**
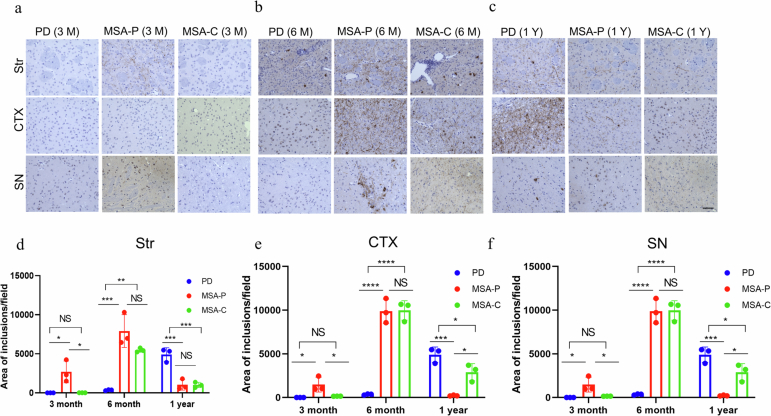
α-synuclein seeds derived from the serum of PD and MSA cases induce different propagation processes of phosphorylated α-synuclein in the injected mouse brain. α-synuclein seeds derived from the serum of PD and MSA cases were injected into the mouse brain striatum. Phosphorylated α-synuclein pathology observed at **(a)** three months (n = 3), **(b)** six months (n = 3), and **(c)** 12 months (n = 3) after the injection. Immunostaining was conducted on each brain region (Str, CTX, and SN) using the anti-p-syn #64 antibody. Scale bars are 50 µm. The total area of the phosphorylated α-synuclein-positive inclusions was quantified chronologically for contralateral Str **(d)** (3months: PD *vs*. MSA-P, p = 0.0154; MSA-P *vs*. MSA-C, p = 0.0158; PD *vs*. MSA-C, p = 0.9997; 6 months: PD *vs*. MSA-P, p = 0.0007; MSA-P *vs*. MSA-C, p = 0.1087; PD *vs*. MSA-C, p = 0.0052, 1 year: PD *vs*. MSA-P, p = 0.0009; MSA-P *vs*. MSA-C, p = 0.9995; PD *vs*. MSA-C, p = 0.0009), contralateral CTX **(e)** (3months: PD *vs*. MSA-P, p = 0.0238; MSA-P *vs*. MSA-C, p = 0.0371; PD *vs*. MSA-C, p = 0.9259; 6 months: PD *vs*. MSA-P, p < 0.0001; MSA-P *vs*. MSA-C, p = 0.9940; PD *vs*. MSA-C, p < 0.0001, 1 year: PD *vs*. MSA-P, p = 0.0007; MSA-P *vs*. MSA-C, p = 0.0117; PD *vs*. MSA-C, p = 0.0405), and ipsilateral SN **(f)** (3months: PD *vs*. MSA-P, p < 0.0001; MSA-P *vs*. MSA-C, p = 0.7635; PD *vs*. MSA-C, p < 0.0001; 6 months: PD *vs*. MSA-P, p = 0.0001; MSA-P *vs*. MSA-C, p = 0.0383; PD *vs*. MSA-C, p = 0.0001, 1 year: PD *vs*. MSA-P, p < 0.0001; MSA-P *vs*. MSA-C, p = 0.0078; PD *vs*. MSA-C, p = 0.0002). Horizontal axis: time after α-synuclein seed injection; vertical axis: total area of phosphorylated α-synuclein-positive inclusions (μm2) per unit area (mm2). Data are represented as mean area per region ± SEM (n = 5 mice per group). Statistical analysis was conducted using two-sided one-way analysis of variance with post hoc Bonferroni test, resulting in significances of p < 0.0001 (****), p < 0.001 (***), p < 0.01 (**), and p < 0.05 (*) among each group. CTX, cortex; MSA, multiple system atrophy; MSA-C, MSA cerebellar variant; MSA-P, MSA Parkinsonian variant; PD, Parkinson’s disease; SN, substantia nigra; Str, striatum.

**Extended Data Fig. 4:**
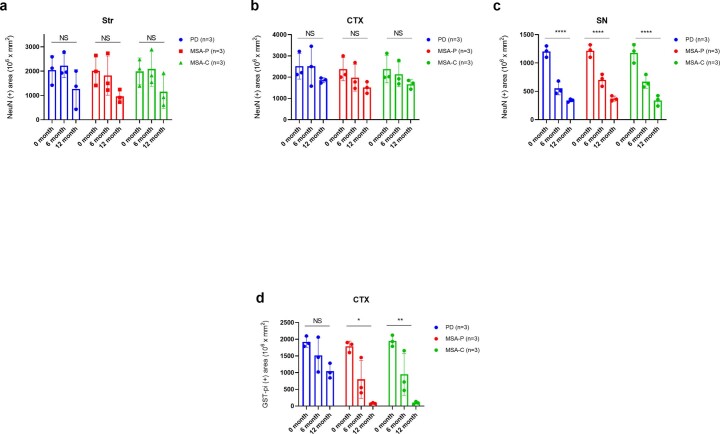
MSA-seeds induce oligodendrocyte degeneration compared with PD-seeds in the injected mouse brains. α-synuclein seeds derived from the serum of PD or MSA cases were injected into the mouse striatum. The total area of the NeuN-positive neurons was quantified chronologically for **(a)** Str (n = 3: PD, p = 0.2282; MSA-P, p = 0.1409; MSA-C, p = 0.2306), **(b)** CTX (n = 3: PD, p = 0.411; MSA-P, p = 0.1465; MSA-C, p = 0.1953), and **(c)** SN (n = 3: PD, p < 0.0001; MSA-P, p < 0.0001; MSA-C, p < 0.0001). The total area of the Gst-pi-positive oligodendrocytes was quantified chronologically for **(d)** CTX (n = 3: PD, p = 0.053; MSA-P, p = 0.017; MSA-C, p = 0.003). Horizontal axis: time after α-synuclein seed injection; vertical axis: total area of NeuN-positive neurons or GST-pi-positive oligodendrocytes (μm2) per unit area (mm2). Data are represented as mean area per region ± SEM, n = 5 mice per group. Statistical analysis was conducted using two-sided one-way analysis of variance, resulting in significances of p < 0.0001 (****), p < 0.01 (**) and p < 0.05 (*) among each group. CTX, cortex; MSA, multiple system atrophy; MSA-C, MSA cerebellar variant; MSA-P, MSA Parkinsonian variant; NS, not significant; PD, Parkinson’s disease; SN, substantia nigra; Str, striatum.

We further confirmed the propagation properties of IR/RT-QuIC-amplified α-synuclein seeds in vivo (Extended Data Fig. 3). We injected PD seeds into the right striatum of mice and observed the gradual propagation accumulation of phosphorylated α-synuclein deposits between 3 months and 1 year as in our previous study^29,30^. MSA seed injection resulted in a more rapid development of phosphorylated α-synuclein pathology by 6 months than PD seed injection. Intriguingly, a reduction in the density of phosphorylated α-synuclein deposits was observed 1 year after MSA seed injection. The significant loss of GST-pi-positive oligodendrocytes in mouse brains 1 year after MSA seed injection (Extended Data Fig. 4) suggests that MSA seeds may induce oligodendrocyte degeneration. In addition, the brain and serum samples of the mice at the sixth and twelfth months after striatal injection of α-synuclein seeds derived from PD and MSA sera were evaluated by IP/RT-QuIC and TEM. The results of IP/RT-QuIC of the brain samples were all positive (Supplementary Fig. 6a and Supplementary Table 12a), and the forming rate was over 1,000 relative fluorescence units (RFU) per hour. TEM revealed that the amplified fibrils derived from the striatum of MSA-seed-injected mice formed MSA-type filaments and MSA-type inclusions as expected. In contrast, the striatum-derived fibrils from PD-seed-injected mice showed not only PD-type but also MSA-type structures in TEM (Supplementary Fig. 6d–g). α-Synuclein seeds were not detected in any sera from seed-injected model mice (Supplementary Fig. 6a and Supplementary Table 12b).

## Discussion

In this study, we proved that the sera from synucleinopathies have the ability to seed, and the detection of α-synuclein seeds amplified from sera by IP/RT-QuIC is a high-performance biomarker to diagnose synucleinopathies. Furthermore, we demonstrated that the structures and propagation properties of the amplified α-synuclein fibrils of LB diseases or MSA differ from each other. These results indicate that the disease-associated α-synuclein seeds exist in the serum of patients with synucleinopathies. Furthermore, the differences in their structures might cause disease-specific phenotypes.

Recently, Kluge et al.^21^ described α-synuclein seeds associated with plasma neuron-derived exosomes as biomarkers of PD. Although the anti-NCAM-1 antibody was employed for purifying the neuron-derived exosomes, there is controversy about whether NCAM-1 is associated with neuron-derived exosomes. Thus, it remains unclear how the α-synuclein seeds detected by Kluge et al. are involved in PD pathomechanisms. Additionally, two groups used nasal mucosa or CSF to demonstrate RT-QuIC utility in synucleinopathy, but these studies did not confirm the structure and properties of α-synuclein seeds^26,27^. Our protocol is simple because it targets the pure synuclein seeds in the serum. We also revealed the pathological impact by structural analyses based on TEM and the cell-based assay and by exploring pathogenic properties using mouse models.

Of the 128 controls, only 11 had α-synuclein seeds in the serum, and most of them were approximately 80 years old. A previous study showed that the incidence of PD increased between 60 years and 89 years of age^31^, and it is well known that aging is one of the important pathogenic factors in PD. IP/RT-QuIC positivity rates in control cases increased with age (Supplementary Table 7), suggesting that these individuals may have incidental LB disease and might develop PD or DLB in the future. Indeed, the structures of α-synuclein fibrils from the controls were similar to those of the PD-type filament.

The differences in sensitivity rates of IP/RT-QuIC observed in the external cohort study may be attributed to the specificities of the PD patient cohorts from different centers when compared to the Luxembourg Parkinson’s Study. Cohorts recruited from tertiary referral centers may differ from population-based cohort studies in various clinical aspects. The Luxembourg Parkinson’s Study recruited individuals with PD from primary and secondary care centers without a university hospital, making this study closer to a population-based approach. As a result, the clinical heterogeneity of patients with PD recruited in Luxembourg is expected to be higher, as reflected by the age at disease onset of patients with PD. One specific feature of the Luxembourg Parkinson’s Study is its longitudinal design with annual follow-up visits for up to 8 years^32^. This follow-up approach enabled regular re-assessment and validation of clinical diagnoses over the course of the disease, which, in some cases, demonstrated a conversion from typical PD to other forms of parkinsonism. Indeed, the external cohort study included cases with a variety of atypical symptoms of idiopathic PD, including young onset PD (age at onset: 35 years), early onset with hemiparkinsonism-hemiatrophy syndrome and parkinsonism with an uncertain diagnosis due to death during the follow-up period. These factors may have contributed to the lower sensitivity of IP/RT-QuIC observed in the external cohort study.

Furthermore, in a recent study, α-synuclein aggregates were detected by protein misfolding cyclic amplification in the CSF of two control patients who subsequently developed symptoms of PD (3%)^33^. It is relevant that previous RT-QuIC studies have also detected aggregates from CSF in the prodromal phases of synucleinopathy^5,34^. In our study, four of nine (44%) patients with RBD showed positive IP/RT-QuIC results, which is lower than that in previous studies, including CSF^5,35^, plasma neuronal exosome^36^, skin biopsy and olfactory mucosa^25^ in patients with idiopathic RBD^37,38^. However, considering the strong positive correlation between disease duration and forming rate of α-synuclein seeds in PD, longer periods of α-synuclein deposition might be important for detecting seeds in serum. In support of this hypothesis, the disease duration of the RBD cases with positive IP/RT-QuIC results was over 6 years. One of the patients with RBD with positive IP/RT-QuIC was converted to PD 2 years after the examination. Furthermore, in the nine patients with RBD, IP/RT-QuIC parameters—including forming rate, *T*_1/2_, *T*_max_ and AUC—were correlated with specific binding ratio, and the forming rate was also correlated with the duration of RBD (Supplementary Table 13). These findings suggest that the positive IP/RT-QuIC might provide a clue for elucidating the phenoconversion of RBD to early PD. However, Poggiolini et al.^35^ reported controversial data, showing no evidence that CSF-derived RT-QuIC positiveness was related to a risk of conversion in RBD cohorts. In this context, a large longitudinal study of serum IP/RT-QuIC from RBD cases is required.

We also found α-synuclein seeds in patients with AD. Recent pathological studies have noted that mixed pathologies with Aβ, tau and α-synuclein may be common in AD pathology^39–41^. However, we could not detect α-synuclein seeds in *PRKN*-positive patients. Most *PRKN*-positive patients had neuronal loss in the substantia nigra compacta without LB pathology, and only six *PRKN*-positive patients with pathologically proven LB were reported^42^; our results support that most *PRKN*-positive patients might not have significant systemic α-synuclein pathology. Consistent with our results, skin biopsy in *PRKN*-positive patients revealed that they had no substantial intraneuronal α-synuclein deposition in sympathetic noradrenergic nerves^43^.

In PD, α-synuclein aggregates in the systemic peripheral autonomic nerves, including the cardiac sympathetic and gastrointestinal nerves, have been reported^9^. It is well known that the ^123^I-MIBG cardiac scintigraphy H/M ratio is a specific diagnostic biomarker of PD. Considering the correlation between the ^123^I-MIBG cardiac scintigraphy H/M ratio and α-synuclein seeds, IP/RT-QuIC may be useful as an alternative marker for ^123^I-MIBG cardiac scintigraphy. As the peripheral-vein-administered or orally administered α-synuclein fibrils penetrated the brain of the mouse model^44^, the pathological α-synuclein seeds, which might originate from the central and/or peripheral nervous system, circulated systemically via both the CSF and blood, resulting in multifocal dissemination of synucleinopathies. The positivity rates were different in the serum and CSF of MSA in our study. However, the serum and CSF α-synuclein seeds in both PD and MSA showed the same structure, suggesting the multifocal dissemination hypothesis.

We observed different serum IP/RT-QuIC-positive rates between PD and MSA. Previous CSF RT-QuIC studies showed that the sensitivity for MSA was also lower than for other synucleinopathies^1,19^. Although these studies analyzed CSF samples, the data were consistent with our results. In contrast, Poggiolini et al.^35^ reported no difference in CSF RT-QuIC positivity between PD and MSA and between MSA-P and MSA-C^35^.

Furthermore, a previous report showed that RT-QuIC could distinguish patients with MSA-P from those with MSA-C^27^, although we did not find a morphological difference between the two phenotypes. This discrepancy might be caused by different samples and protocols. Detecting the serum α-synuclein seeds derived from MSA by our IP/RT-QuIC method might be slightly difficult. Furthermore, a correlation was not found between the serum α-synuclein seed detection in MSA and clinical phenotypes (such as parkinsonism), cerebellar ataxia, disease duration and disease severity. These findings might indicate a lower concentration of serum α-synuclein seeds in patients with MSA than in patients with PD. Thus, we need to improve the detection sensitivity of IP/RT-QuIC to detect a smaller amount of serum α-synuclein seeds.

Recent studies have demonstrated that seeds are present in the CSF of patients with PD or MSA as distinct conformational strains^2^. Consistent with these results, our TEM study and cell-based intracellular inclusion assay also demonstrated clear morphological differences between PD seeds and MSA seeds. However, the morphological features of MSA fibers amplified from patient brain tissue observed by TEM, as shown in a previous report^44^, seemed to be different from those of serum-derived fibrils. Although MSA fibrils amplified by serum IP/RT-QuIC may differ in morphology from MSA brain-derived fibrils, the fibrils in serum could be distinguished from PD disease-derived fibrils because they maintained disease-specific structures.

Finally, we confirmed that the amplified fibrils from serum have prion-like propagation ability in vivo. After 6 months, the MSA seeds propagated more rapidly than the PD seeds in regions with direct and indirect connections to the injected site. Six months after seed inoculation, we revealed a significantly larger deposited area of phosphorylated MSA seeds than that of PD seeds. However, 1 year after the inoculation, the aggregation regions induced by MSA seeds were reduced. We also observed a significant loss of GST-pi-positive oligodendrocytes in mouse brains 1 year after MSA seed inoculation (Extended Data Fig. 4). It is probable that MSA seeds rapidly propagate through cell division of oligodendrocytes and induce their degeneration before neuronal cell death. In the future, the association between the systemic α-synuclein seed structure and neuronal degeneration with or without oligodendrogliopathy in synucleinopathy should be investigated. Recently, it was reported that cryo-electron microscopy structures of in vitro amplified MSA fibrils did not necessarily replicate those of the seeds, even when using MSA brain-derived filament preparations to seed in vitro assembly^45^. The amplified fibrils from the LB disease and MSA sera induced different forms of inclusions in cultured cells and showed different propagation processes in the mouse brain, suggesting that the fibrils amplified by IP/RT-QuIC preserve their distinct structural and functional properties to distinguish between LB disease and MSA.

IP/RT-QuIC could amplify the α-synuclein seeds from the brain of the seed injection mouse model; however, it should be noted that the seeds were not detected in the serum of the mouse model (Supplementary Fig. 6a and Supplementary Table 12b). Because we used 0.1 ml of sera from the patients for serum IP/RT-QuIC, we did not obtain a sufficient amount of blood sample from the mouse model for detecting α-synuclein seeds. The TEM analysis revealed that the amplified fibrils derived from the striatum of MSA-seed-injected mice formed MSA-type filaments and MSA-type inclusions as expected. In contrast, the striatum-derived fibrils from PD-seed-injected mice showed not only PD-type but also MSA-type structures in TEM (Supplementary Fig. 6d–g). Although the reason for the difference in morphologies between α-synuclein seeds derived from the PD seed injection mouse model and the original patient seeds remains unclear, the conditions of IP/RT-QuIC for detecting α-synuclein seeds derived from mouse brain might be different than those for human serum. This study has several limitations. Almost all participants’ diagnoses were based on clinicoradiological features without neuropathological confirmation. We also did not examine the CSF for all participants. Therefore, we could not rule out that some patients had false clinical diagnoses. Nonetheless, the IP/RT-QuIC assay provides the possibility of future applications as a biomarker in clinical trials and personalized medicine for synucleinopathies.

## Methods

### Study design and participants

We enrolled patients with PD, MSA, DLB, RBD, PSP, AD and PD with *PRKN* mutations (Supplementary Table 14). The diagnosis was based on standard criteria. Control group participants without any neurodegenerative disorders were also recruited from the Juntendo University Department of Neurology and Neurosurgery. To validate the results obtained from this cohort, samples were obtained from previously reported cohorts (the second cohort)^28^, and an external cohort from the University of Luxembourg (Extended Data Table 1) was examined. This study was approved by the Ethics Committee of Juntendo University (no. 2021100) and the National Ethics Board (CNER ref: 201407/13) and the Data Protection Committee (CNPD ref: 446/2017) of the University of Luxembourg. Written informed consent was obtained from all participants before enrollment. The study procedures were performed in accordance with the Declaration of Helsinki. The diagnostic criteria for PD, MSA, DLB, PSP, AD and RBD were based on the Movement Disorder Society (MDS)-sponsored PD clinical criteria^46^, Gilman’s criteria^47^, definitions and guidelines provided by the DLB Consortium^48^, MDS-sponsored PSP clinical criteria^49^ and the National Institute of Neurological and Communicative Disorders and Stroke and the Alzheimer’s Disease and Related Disorders Association^50^, respectively. Patients with RBD were polysomnographically examined.

The following clinical data were collected: age, sex, disease duration, total levodopa equivalent daily dose^51^, Hoehn–Yahr scale score and MDS Unified Parkinson’s Disease Rating Scale (MDS-UPDRS) Part III score determined in the participant’s ‘ON’ state and Mini-Mental State Examination (MMSE) score. Both serum and CSF data were available from nine patients (six PD and three MSA) and 35 controls (Extended Data Table 6).

#### ^123^I-MIBG cardiac scintigraphy and ^123^I-ioflupane single-photon emission tomography

^123^I-MIBG cardiac scintigraphy and ^123^I-ioflupane single-photon emission tomography (DaT Scan) were performed to assess any cardiac sympathetic denervation in the heart and nigrostriatal dopaminergic neurodegeneration, respectively. We used the same methodology as previously described^52,53^.

#### Sample collection

Laboratory procedures included blood sampling, and, in a subset of participants who provided consent for the procedure, a lumbar puncture was performed on the same day as the blood sampling. Blood sampling was performed between 9:00 and 12:00. There were no restrictions, such as fasting. Patients with a history of cancer were excluded from the study. To rule out PDD and DLB, patients with PD with an MMSE score of ≤23 points were excluded. Three specialized neurologists confirmed each diagnosis (T.H., A.O. and S.U.).

Serum samples were collected, processed, aliquoted and frozen at −80 °C according to standardized procedures. CSF samples were obtained from six patients with PD, three patients with MSA and 20 controls. CSF samples were collected using polypropylene tubes after lumbar puncture at the L4/L5 or L3/L4 interspace with atraumatic needles. The samples were centrifuged at 3,000*g* for 15 min at 4 °C, aliquoted and stored at −80 °C until analysis. The methodology of sample collection was approved by the institutional review board of the study center (Juntendo University), and all study participants provided written informed consent.

### Procedures

#### Purification of recombinant α-synuclein

Recombinant human α-synuclein protein was purified from *Escherichia coli* BL21 harboring pRK172-α-synuclein (Y136-TAT) as previously reported^54^. The protein was filtered through a 0.22-μm filter (Merck) and dialyzed using a 10-kDa molecular weight cutoff (MWCO) dialysis membrane (Thermo Fisher Scientific) against 30 mM Tris-HCl, pH 7.5, and cleared using a 20-min centrifugation at 113,000*g*. Protein concentration was measured using the Pierce BCA Protein Assay Kit (Thermo Fisher Scientific). The purified protein sample was kept at −80 °C.

#### Preparation of pre-formed fibrils

Purified α-synuclein monomers (100 μM, 150 μl) were incubated at 37 °C in a shaking incubator (Eppendorf) at 1,200 r.p.m. in a solution of 50 mM Tris-HCl containing 100 mM NaCl (pH 8.0) for 5 d. Turbidity measurements were performed at OD_600_. After 5 d, α-synuclein pre-formed fibrils (PFFs) were pelleted by spinning at 50,000*g* for 20 min and suspended in PBS.

#### IP

As shown in Supplementary Fig. 7, 100 µl of IP lysis buffer (1% BSA, 150 mM NaCl, 1% Triton X, 50 mM Tris HCl, pH 7) containing 1.7 µg of MJFR-1 (anti-α-synuclein antibody, Abcam) and 30 µl of protein A/G magnetic beads (Thermo Fisher Scientific) were incubated overnight at 4 °C. Then, 100 µl of serum (1 mg of protein per milliliter) was added to the buffer and rotated at 4 °C for 2 h. The proteins were eluted using 20 µl of 50 mM glycine, and the samples were adjusted to pH 7.5.

We confirmed that MJFR-1 could detect mouse α-synuclein monomers and fibrils by dot blot and western blotting. Mouse serum was then immunoprecipitated with MJFR-1, and IP/RT-QuIC was performed (Supplementary Fig. 6b,c).

#### RT-QuIC

As shown in Supplementary Fig. 7, the RT-QuIC assay was modified according to a previously reported protocol^16,55^. The reaction buffer (RB) contained 100 mM phosphate buffer (pH 7.5–8.0), 10 μM thioflavin T (ThT), 0–170 mM NaCl and 0.1 mg ml^−1^ recombinant α-synuclein. Each well of a black 96-well plate with a clear bottom (Thermo Fisher Scientific) contained 95 μl of RB and 37 ± 3 mg of 0.5-mm zirconium/silica beads (Thermo Fisher Scientific). Reactions were seeded with 5 μl of IP product solution from the serum to a final reaction volume of 100 μl. The plates were incubated in a FLUOstar OPTIMA microplate reader (BMG Labtech) at 30 °C for 120 h with intermittent shaking cycles: double-orbital with 1 min of shaking at 200 r.p.m., followed by 14 min of rest. ThT fluorescence measurements (450 nm excitation and 480 nm emission) were taken every 15 min^24^. RT-QuIC for CSF α-synuclein was performed in the same manner as RT-QuIC for a serum sample but without IP. Each sample was run in triplicate. A positive response was defined as an RFU value of more than 260,000 at 120 h. Positive signals in two or more of the triplicate wells were considered positive (Supplementary Fig. 8). If the RFU value did not reach 260,000 within 120 h, it was considered negative. The pH of the reaction buffer was adjusted to 7.2–7.8.

For each IP/RT-QuIC analysis, we prepared six serial dilution series (10 ng µl^−1^, 1 ng µl^−1^, 0.1 ng µl^−1^, 0.01 ng µl^−1^, 0.001 ng µl^−1^ and 0 ng µl^−1^ as final concentrations) using pre-formed α-synuclein fibrils (PFFs) as the positive control, and a standard curve was generated to estimate the concentration of seeds in the serum. To increase the reliability of each RT-QuIC experiment, we validated the experiment confirming whether the standard curve shows a concentration-dependent increase in fluorescence intensity: 10 ng µl^−1^ to 0.01 ng µl^−1^ of PFF eventually reaches the RFU value of 260,000 within 120 h; controls without PFF, such as the negative control, do not reach 260,000 RFU, not showing even a slight increase; and 0.001 ng µl^−1^ may or may not reach 260,000 RFU (Supplementary Figs. 8 and 9). When an experiment did not satisfy the criteria, we considered that the result was unreliable, and the experiment was re-performed as a new experiment (Supplementary Figs. 8 and 9). The actual fluorescence curves of an assay (both positive and negative samples) are shown in Supplementary Fig. 8 as a representative of our criteria to judge the experiment reliability. Each IP sample was analyzed in triplicate by RT-QuIC, and the results were validated to determine whether the RFU value reaches 260,000 within 120 h. A positive response was defined as a RFU value of ≥260,000 at 120 h, and positive signals in two or more of the triplicate wells were considered positive.

#### TEM

TEM images were obtained at the Laboratory of Morphology and Image Analysis of Juntendo University. Products of IP/RT-QuIC were assessed using an HT7700 TEM (Hitachi) after adsorption onto copper grids and negative staining with 2% uranyl acetate.

#### Analysis of the assay reproducibility

Reliability and reproducibility were assessed using kappa statistics. The kappa coefficient is the percentage of instances of agreement with the likelihood of agreement based on chance alone. A kappa coefficient of 1.00 indicates perfect agreement. Results with increased fluorescence up to 260,000 RFU were considered positive and are listed in Supplementary Table 1 by the number of positive wells in each IP sample in the first cohort; for example, two positives and one negative are shown as 2/3. For synucleinopathies, 35.9% of IP samples from patient sera showed a full (3/3) positive response, and 54.5% showed 2/3, indicating that 90.4% of patients with synucleinopathies were correctly distinguished (Supplementary Table 1). Furthermore, to verify our IP/RT-QuIC methodology, we repeated the IP/RT-QuIC analysis against previously analyzed samples, including 18 cases of PD, 17 cases of MSA and 46 healthy controls, and we compared the number of positives in each experiment, as shown in Supplementary Table 2. The reproducibility of each experiment was evaluated by the kappa coefficient. The variation of the kappa coefficient for PD, MSA and control (CTRL) were 0.89, 1.00 and 0.83, respectively; intra-batch reproducibility–1. Positive/negative concordance rates for IP/RT-QuIC were also analyzed using serum samples collected from the same cases at different dates and times; intra-batch reproducibility–2. The kappa coefficient of positive/negative determination was 1.00, and the concordance rate for each well was 0.71 (Supplementary Table 3). As the inter-batch reproducibility–1, we further determined the concordance rates for the result determination (three independent examiners; A.O., T.H. and S.U.) (Supplementary Table 4) and IP/RT-QuIC technique; inter-batch reproducibility–2 (two independent examiners; A.O. and H.K.) (Supplementary Table 5). The kappa coefficient of the inter-batch reproducibility–1 was 0.79 (evaluator A versus evaluator B), 0.97 (evaluator B versus evaluator C) and 0.82 (evaluator A versus evaluator C), and the inter-batch reproducibility–2 was 0.68. These reproducibility results represent that our IP/RT-QuIC analysis is highly reliable. We conducted TEM analysis for α-synuclein fibrils that were amplified by IP/RT-QuIC from sera of patients with pathologically confirmed synucleinopathies (three PD and one MSA from the internal cohort and one PDD from the external cohort) to analyze the assay reproducibility. In the first cohort, the primary morphologies derived from PD seeds and PDD seeds were paired filaments or bundled multiple filaments, whereas those from MSA seeds were straight filaments (Supplementary Fig. 10). The single filament of LB diseases had narrower widths than those of MSA seeds.

#### Cell seeding assay

Human embryonic kidney (HEK) 293 cells were stably transfected with the C-terminally EGFP-fused A53T mutated human α-synuclein plasmid (phaSyn-GFP A53T). The HEK293-SynG A53T cell line was selected with 500 µg ml^−1^ G418 and subcloned. The cells were maintained in DMEM with 10% FBS, 500 µg ml^−1^ G418 and 1% penicillin–streptomycin and incubated in 5% CO_2_ at 37 °C. For visualization studies of α-synuclein aggregation in cells, HEK293-SynG A53T cells were grown on coverslips coated with poly-l-lysine (Merck) in 24-well plates at 1 × 10^4^ cells per well. The α-synuclein seeds amplified from patients’ serum using IP/RT-QuIC were sonicated in a water bath sonicator for 1 min, and 2 μl of α-synuclein seeds was subjected to transduction using Lipofectamine 3000 reagent (Thermo Fisher Scientific). After 48 h of transduction, cells were fixed in neutralized formaldehyde (Wako) and blocked with 1% FBS and 0.1% Triton X-100 in PBS and a protease inhibitor cocktail (Merck). Fixed cells were mounted in ProLong Diamond Antifade Mountant (Thermo Fisher Scientific) after DAPI staining. Super-resolution structured illumination microscopy (SR-SIM) was performed using a Zeiss Elyra super-resolution microscope equipped with a ×100 oil immersion objective lens (NA 1.46, Carl Zeiss). A whole-cell *z-*stack (each slice = 0.11 μm) was acquired with three rotations and analyzed to reconstruct super-resolution images. A maximum projection was created. All images were processed using Zen (Carl Zeiss) and ImageJ64 (NIH Image). For quantitative analysis of α-synuclein aggregate density, whole-cell images were taken with a BZ-X800 (Keyence) using a ×63 oil immersion objective lens and BZ-H4XF/sectioning module. The fluorescence intensity of α-synuclein inclusion bodies and the area of inclusion bodies were measured and analyzed by Hybrid Cell Count software (Keyence).

#### Analysis of the assay reproducibility of the cell seeding assay

We analyzed the morphology of intracellular α-synuclein inclusions by super-resolution microscopy using IP/RT-QuIC-amplified α-synuclein seeds from 20 PD, 20 MSA, 5 DLB and 10 CTRL cases in the first cohort. For the analysis of the fluorescence density of the intracellular inclusion bodies, we confirmed 100 cells per case using Hybrid Cell Count software. The morphology was also evaluated in a blinded manner by three examiners (T.H., A.O. and G.M.) from different institutes; the results are shown in Supplementary Table 11. The agreement of disease diagnosis based on the pathology or inclusion body morphology ranged from 60% to 100%, with a kappa coefficient of 0.75 (first trial evaluator A versus evaluator B) and 1.0 (second trial evaluator B versus evaluator C) (inter-batch reproducibility; Supplementary Table 11), and the patient-to-patient reproducibility (intra-batch reproducibility; Supplementary Table 11) had a kappa coefficient of 0.84.

#### Animal model

C57BL/6J mice were obtained from CLEA Japan. All breeding, housing and experimental procedures were performed in accordance with the guidelines for animal care of Juntendo University and approved by the Juntendo University Animal Care and Use Committee (approval no. 310187). Only male mice were used in this study. We sonicated the RT-QuIC products before the intracerebral injection (using Bioruptor UC100-D2: Cosmo Bio: TOS; 20 pulses; each pulse consisting of a 20-s ‘ON’ period and a 20-s ‘OFF’ period). Mice 2–3 months of age were anesthetized using an isoflurane/oxygen/nitrogen mixture and unilaterally injected with 10 μg of products of RT-QuIC into the right striatum (A-P: 0.2 mm; M-L: +2.3 mm; D-V: −2.6 mm, from bregma) using a 10-μl Hamilton syringe at a rate of 0.1 μl min^−1^. The mice were anesthetized with an isoflurane/oxygen/nitrogen mixture and killed by decapitation at various pre-determined timepoints (3 months, 6 months and 1 year). For histological studies, mice were perfused with PBS, followed by 4% paraformaldehyde (PFA) in PBS, followed by overnight incubation of the tissue after fixation in either neutral buffered formalin (Thermo Fisher Scientific) or 70% ethanol before undergoing processing and embedding in paraffin.

#### Tissue preparation

Mice were perfused with PBS, followed by 4% PFA in PBS. Brains were post-fixed, dehydrated and embedded in paraffin wax to prepare paraffin sections. Sections of 5-μm thickness were cut using an HM430 sliding microtome (Leica).

#### Immunohistochemistry

Autoclaved paraffin sections were incubated with a blocking solution containing 5% skim milk in TBST (20 mM Tris-HCl, pH 8.0, 150 mM NaCl, 0.05% Tween 20) for 1 h. Sections were incubated with the primary antibody (anti-p-syn antibody (mouse monoclonal, pSyn #64, 1:300, FUJIFILM Wako), neuronal marker (rabbit polyclonal, anti-NeuN, 1:500, ab104225, Abcam), oligodendrocyte marker (rabbit polyclonal, anti-GST-pi, 1:500, 312, MBL)) in TBST overnight at 4 °C, followed by incubation with the secondary antibody biotinylated anti-rabbit IgG (1:300, BA1000, Vector Labs) and biotinylated anti-mouse IgG (1:300, BA9200, Vector Labs). For diaminobenzidine staining, sections were quenched with 3% H_2_O_2_/methanol for 30 min before blocking and incubated with the VECTASTAIN Elite ABC Kit reagent (Vector Labs) for 30 min after secondary antibody incubation. Color development was performed using 3,3-diaminobenzidine/H_2_O_2_. To analyze the density of aggregation (p-syn) inclusions, whole-brain sections were imaged with a Keyence microscope using bright-field capture. Multiple fields were captured using a ×10 objective and stitched together using the Keyence Merge function. The density of α-synuclein aggregates was quantified using Hybrid Cell Count software based on hue.

#### Immunoblotting

Samples were mixed with sodium dodecyl sulfate (SDS) sample buffer. Equal protein amounts were separated by SDS-polyacrylamide gel electrophoresis (SDS-PAGE) after determining protein concentrations by bicinchoninic acid (BCA) assays. Samples were transferred to polyvinylidene difluoride (PVDF) membranes and blocked with Bullet Blocking One (Nacalai) at room temperature (15–25 °C) for 5 min. After blocking, PVDF membranes were incubated overnight with primary antibodies (anti-α-synuclein antibody (rabbit monoclonal, MJFR1, 1:1,000 ab138501, Abcam) and anti-albumin antibody (mouse monoclonal, 1:10,000, PGI 4A1C11)) at 4 °C. The membranes were washed three times with TBST, and secondary antibodies (peroxidase affinipure goat anti-rabbit Ig G (1:10,000, 111-035-144, Jackson ImmunoResearch) and goat anti-human IgG (HRP) pre-adsorbed (1:10,000, ab98624, Abcam)) were added. ECL prime (RPN2232, GE Healthcare) was used for chemiluminescence, and Fusion FX (VILBER) instruments were used for imaging.

#### Dot blot

Resuspended pellets (2.5 µl) were spotted onto nitrocellulose membranes and air dried for 1 h at room temperature (15–25 °C). After blocking with Bullet Blocking One (Nacalai) at room temperature for 5 min and immunostaining, the detection was performed as for western blotting.

#### Analysis of contaminant proteins

We conducted a dot blot of sera and IP/RT-QuIC products to deduce the amount of contaminated serum-derived proteins. In the dot blot, the serum albumin contamination was detected in the IP sample but not in the IP/RT-QuIC products. Serum IP products derived from both patients with PD and controls contained human IgG and the antibody used in the IP procedure. We also performed a Coomassie brilliant blue assay to ensure that there were not large amounts of contaminants in the IP/RT-QuIC products. The related results are shown in Supplementary Fig. 11.

### Statistics

We expected that the lower limit of the 97% CI for the sensitivity was satisfied in 95% of the patients for an estimated sample size of 300 participants (*n* = 235; controls, *n* = 15)^22^.

The primary objective was to assess the diagnostic performance of serum biomarkers in synucleinopathy diagnosis. Numerical variables are expressed as mean ± s.d., median (interquartile range) or mean ± s.e.m.

Baseline characteristics were summarized using standard descriptive statistics, and a descriptive analysis was performed. The chi-squared and Fisher’s exact tests were used to examine categorical variables; the normality of the distribution was determined using the Shapiro–Wilk test and the independent *t*-test; and the Mann–Whitney *U*-test, one-way ANOVA and post hoc Bonferroni test were used to examine continuous variables. Correlations between variables were assessed using Pearson correlation analyses, and the standardized correlation coefficients are presented. From these models, we derived odds ratios for predictors, sensitivity, specificity, related statistics, ROC curves and estimates of the AUCs (AUC = 0.5 indicates no discrimination, and a perfect diagnostic test would have AUC = 1). ROC analysis was performed using the forming rate of patients with synucleinopathy and CTRL. The cutoff value was calculated using Youden’s J index, which was calculated for all points on the ROC curve. Logistic regression analyses assessed, in a more clinically useful manner, biological and demographic variables and their linear combinations in terms of their predictive value for discriminating among diagnostic groups or sets of such groups. The diagnostic accuracy of the IP/RT-QuIC parameters was assessed by the ROC analysis. κ overcomes this issue, as it provides an inter-observer agreement measure between two or more observers when the variable assessed is on a binomial or categorical scale.

All statistical analyses were performed using IBM SPSS (version 22.0), SAS version 9.4 (SAS Institute) and GraphPad Prism version 8.0 (GraphPad Software). The figures were prepared using Prism 8 for Windows OS (GraphPad Software). For all figures, each point represents at least three assays per sample and three technical replicates per assay unless otherwise specified. All statistical tests were two-sided. An alpha level of *P* < 0.05 was used to determine statistical significance. Randomization was not performed in this study.

### Reporting summary

Further information on research design is available in the Nature Portfolio Reporting Summary linked to this article.

## Online content

Any methods, additional references, Nature Portfolio reporting summaries, source data, extended data, supplementary information, acknowledgements, peer review information; details of author contributions and competing interests; and statements of data and code availability are available at 10.1038/s41591-023-02358-9.

## Supplementary information

Supplementary Information Supplementary Figs. 1–11, Tables 1–14 and Methods.

Reporting Summary

## Figures and Tables

**Extended Data Table 1:**
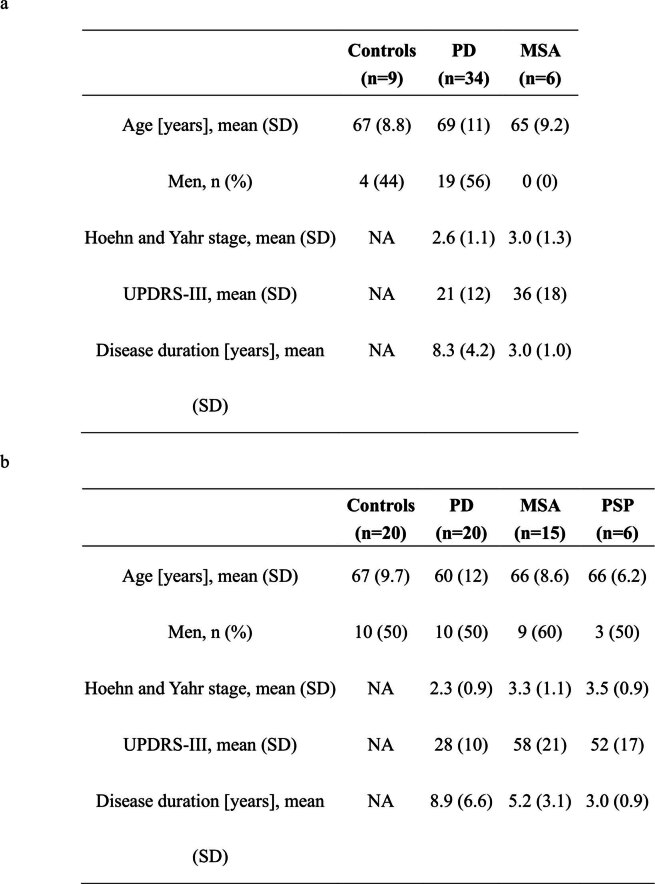
Characteristics of the participants in the second cohort (**a**) and the external cohort (**b**)

NA, not applicable.

**Extended Data Table 2:**
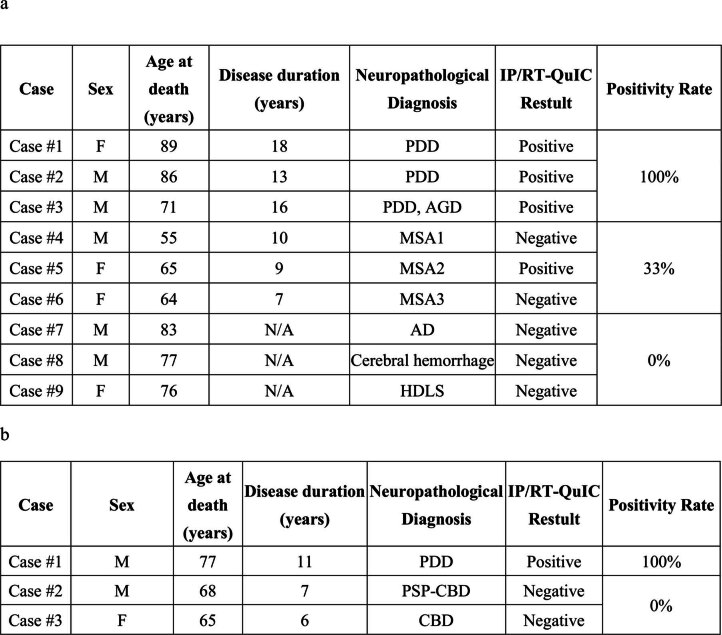
IP/RT-QuIC in pathologically confirmed cases in the internal cohort (**a**) and the external cohort (**b**)

Data are presented as numbers. AGD, argyrophilic grain dementia; CBS, cortical basal syndrome; F, female; HDLS, hereditary diffuse leukoencephalopathy with spheroids; M, male; NA, not applicable.

**Extended Data Table 3:**
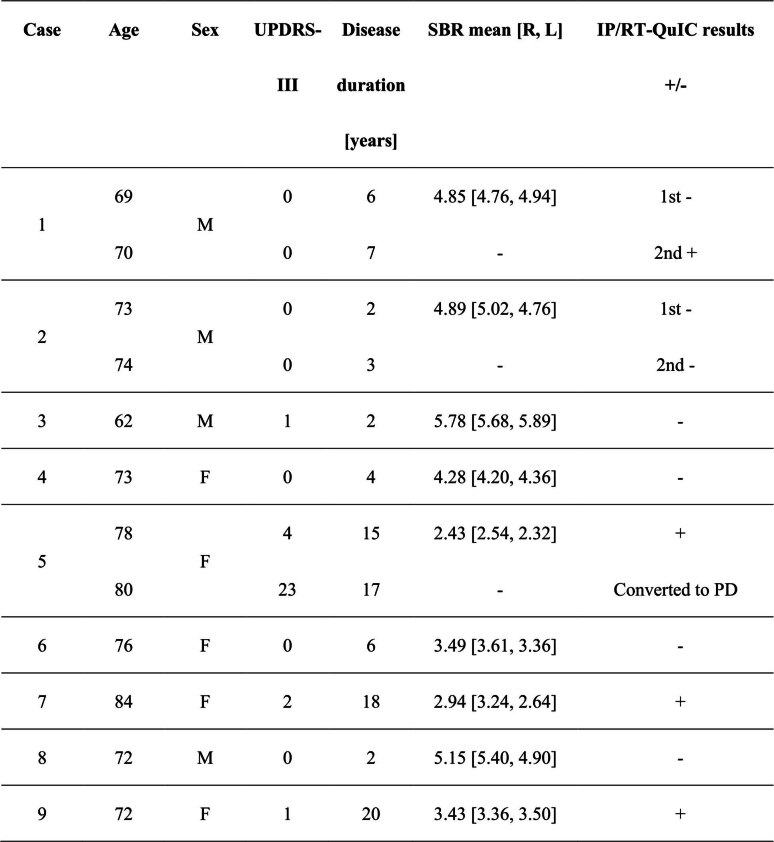
IP/RT-QuIC in RBD

F, female; L, left; M, male; R, right; SBR, specific binding ratio.

**Extended Data Table 4:**
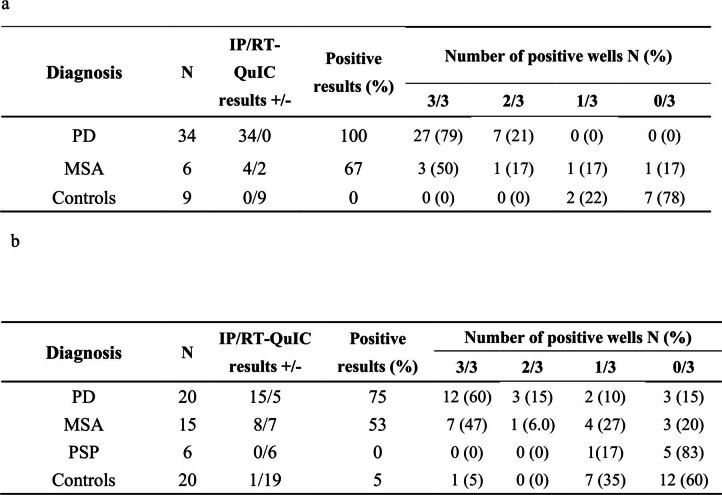
Serum α-synuclein IP/RT-QuIC results of the second cohort (**a**) and the external cohort (**b**)

Data are presented as numbers. *n*, number of participants who received IP/RT-QuIC.

**Extended Data Table 5:**
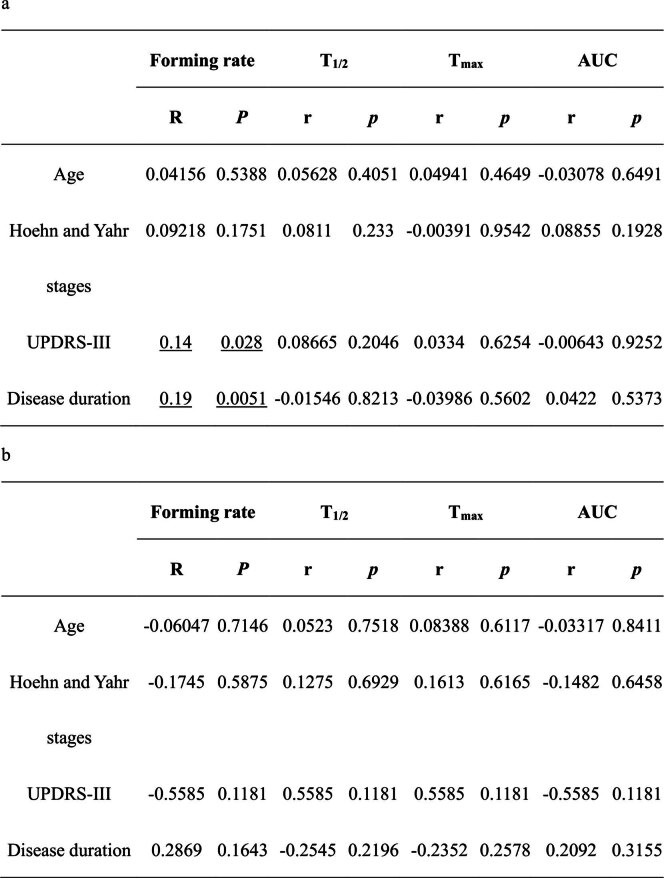
The correlation between IP/RT-QuIC parameters and characteristics and clinical parameters of PD (**a**) and MSA (**b**) cases

Correlations between variables were assessed using two-tailed Pearson correlation analyses. Underlined values indicate *P* < 0.05. T_1/2_, time to reach 130,000 fluorescence; T_max_, time to reach maximum (260,000) fluorescence.

**Extended Data Table 6:**
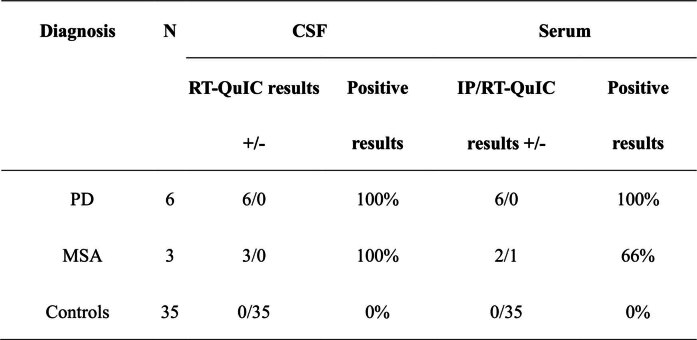
Serum α-synuclein IP/RT-QuIC results and CSF RT-QuIC assay results for participants from whom both serum and CSF samples were obtained

Both serum and CSF data are available from nine patients (six PD and three MSA) and 35 controls. Data are presented as numbers. *n*, number of participants who received RT-QuIC or IP/RT-QuIC.

## Data Availability

The authors declare that all relevant data used to conduct the analyses are available within the article. To protect the privacy and confidentiality of patients in this study, clinical data are not made publicly available in a repository or in the supplementary material of the article but can be requested at any time from the corresponding author. Any requests will be reviewed within a timeframe of 2–3 months by the Ethics Committee of Juntendo University to verify whether the request is subject to any intellectual property or confidentiality obligations. All data shared will be de-identified.
