# Author Correction: Propagative α-synuclein seeds as serum biomarkers for synucleinopathies

**DOI:** 10.1038/s41591-025-03521-0

**Published:** 2025-01-24

**Authors:** Ayami Okuzumi, Taku Hatano, Gen Matsumoto, Shuko Nojiri, Shin-ichi Ueno, Yoko Imamichi-Tatano, Haruka Kimura, Soichiro Kakuta, Akihide Kondo, Takeshi Fukuhara, Yuanzhe Li, Manabu Funayama, Shinji Saiki, Daisuke Taniguchi, Taiji Tsunemi, Deborah McIntyre, Jean-Jacques Gérardy, Michel Mittelbronn, Rejko Kruger, Yasuo Uchiyama, Nobuyuki Nukina, Nobutaka Hattori

**Affiliations:** 1 Department of Neurology, Juntendo University Faculty of Medicine, Tokyo, Japan; 2 Department of Histology and Cell Biology, Nagasaki University School of Medicine, Nagasaki, Japan; 3 Medical Technology Innovation Center, Juntendo University Faculty of Medicine, Tokyo, Japan; 4 Laboratory of Morphology and Image Analysis, Biomedical Research Core Facilities, Juntendo University Faculty of Medicine, Tokyo, Japan; 5 Department of Neurosurgery, Juntendo University Faculty of Medicine, Tokyo, Japan; 6 Neurodegenerative Disorders Collaboration Laboratory, RIKEN Center for Brain Science, Saitama, Japan; 7 Department of Neurology, Institute of Medicine, University of Tsukuba, Tsukuba, Japan; 8 Transversal Translational Medicine, Luxembourg Institute of Health (LIH), Strassen, Luxembourg; 9 Luxembourg National Center of Pathology (NCP), Laboratoire National de Santé (LNS); Department of Cancer Research (DOCR), Luxembourg Institute of Health (LIH); Luxembourg Centre of Neuropathology (LCNP), Luxembourg Centre for Systems Biomedicine (LCSB), Faculty of Science, Technology and Medicine (FSTM) and Department of Life Sciences and Medicine (DLSM), University of Luxembourg, Esch-sur-Alzette, Luxembourg; 10 Centre Hospitalier de Luxembourg (CHL); Translational Neuroscience, Luxembourg Centre for Systems Biomedicine (LCSB), University of Luxembourg, Strassen, Luxembourg; 11 Department of Cellular and Molecular Neuropathology, Juntendo University Faculty of Medicine, Tokyo, Japan; 12 Laboratory of Structural Neuropathology, Graduate School of Brain Science, Doshisha University, Kyoto, Japan

**Keywords:** Parkinson's disease, Cellular neuroscience

Correction to: *Nature Medicine* 10.1038/s41591-023-02358-9, published online 29 May 2023.

In the version of the article initially published, in Extended Data Fig. 3c, the image in the SN row under MSA-C (1 Y) was incorrect and has now been amended in the HTML and PDF versions of the article.

