# Supplementary material for: Propagative α-synuclein seeds as serum biomarkers for synucleinopathies
Source: Nat Med. 2023 May 29;29(6):1448–55. doi: 10.1038/s41591-023-02358-9 (PMC10287557; doi:10.1038/s41591-023-02358-9)
Supplement: Supplementary file 1 — Supplementary Figs. 1–11, Tables 1–14 and Methods. [file 41591_2023_2358_MOESM1_ESM.pdf]

nature medicine

Article https://doi.org/10.1038/s41591-023-02358-9

Propagative α-synuclein seeds as serum biomarkers for synucleinopathies

In the format provided by the authors and unedited

Supplementary Figures and Tables

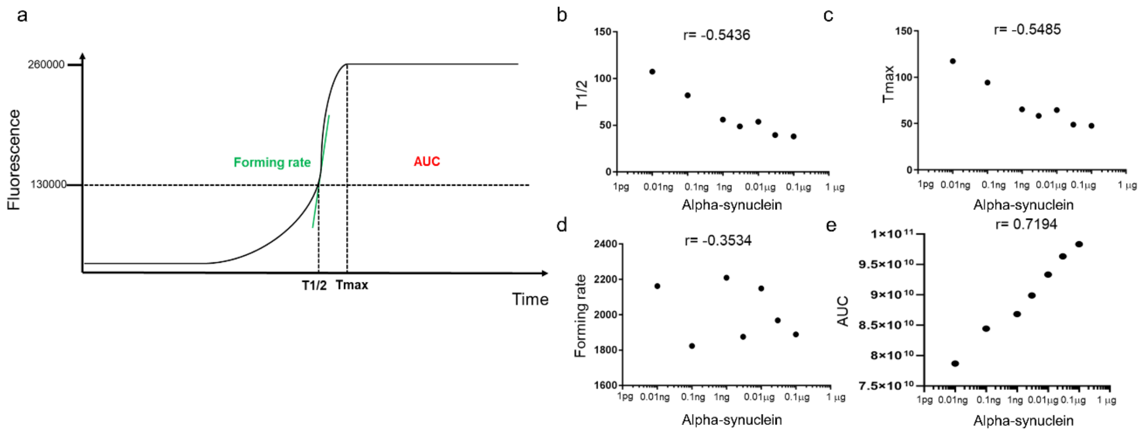

Supplementary Fig. 1. Parameters of α-synuclein amyloid formation with IP/RT-QuIC.

We analyzed 100 μl of eight different concentrations of the recombinant α-synuclein fibril: 10 ng/μL, 1 ng/μL, 0.1 ng/μL, 0.01 ng/μL, 0.001 ng/μL, and 0 ng/μL. We used 10 ng/μL to 0.01 ng/μL of recombinant α-synuclein fibrils as positive controls because these concentrations ensure that the relative fluorescence unit value reaches 260,000. We used 0 ng/μl as the negative control because the relative fluorescence unit value is less than 260,000. A concentration of 0.001 ng/μl (1000 pg/ml) may or may not reach 260,000.

(a) Schematic view of the parameters of serum α-synuclein-IP/RT-QuIC. (b) The time to reach 130k fluorescence ($T_{1/2}$) and (c) the time to reach maximum (260k) fluorescence

(T_{max}) are shortened in a dose-dependent manner. (d) The amyloid formation rate is not dependent on α-synuclein fibrils. The forming rate is represented by the slope of the tangent line at the inflection point in the sigmoid curve of IP/RT-QuIC and indicates the rate of aggregate formation. (e) The area under the curve (AUC) is increased in a dose-dependent manner in α-synuclein fibrils.

IP/RT-QuIC, immunoprecipitation/real-time quaking-induced conversion

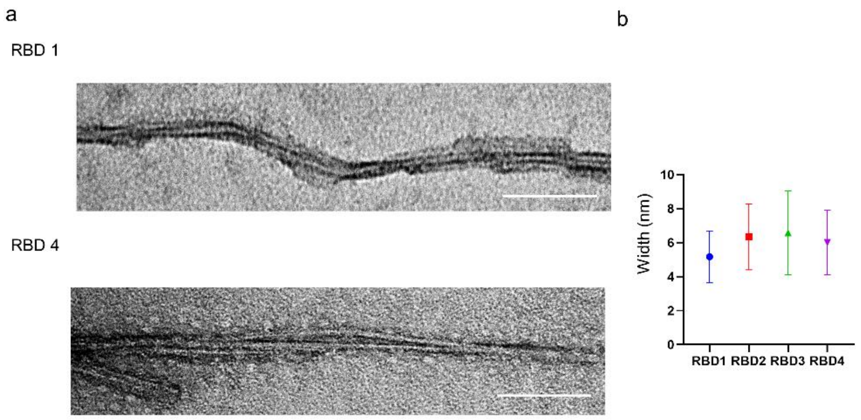

Supplementary Fig. 2. Morphological analysis of amplified products derived from RBD cases with positive IP/RT-QuIC results.

(a) Negative-stained transmission electron microscopy images of α-synuclein fibrils derived from RBD cases. (b) The widths of products derived from RBD cases with positive IP/RT-QuIC results are shown. The data represent mean ± standard error of the mean. We measured the width at two sites per fibril (n=3). Scale bars are 100 nm. RBD, rapid eye movement sleep behavior disorder.

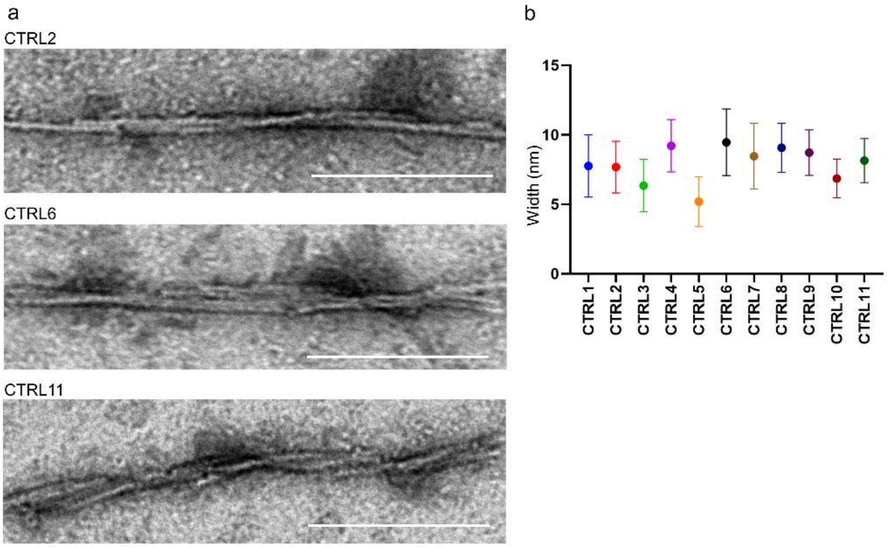

Supplementary Fig. 3. Morphological analysis of amplified products derived from control cases with positive IP/RT-QuIC results.

(a) Negative-stained transmission electron microscopy images of α-synuclein fibrils derived from controls. (b) The widths of products derived from controls with positive IP/RT-QuIC results are shown. The data represent mean ± standard error of the mean. We measured the width at two sites per fibril (n=3). Scale bars are 100 nm.

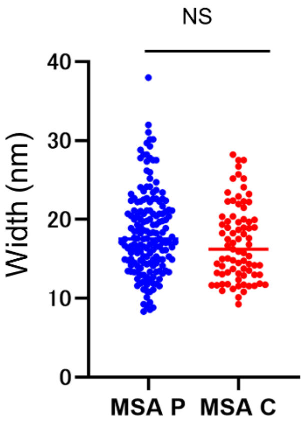

Supplementary Fig. 4. The widths of α-synuclein fibrils derived from MSA-P and MSA-C.

Each dot corresponds to a different fibril. Data are expressed as mean ± standard error of the mean. Statistical analysis was conducted using a two-tailed t-test ($p=0.7089$). MSA-P, $n=26$; MSA-C, $n=13$. We measured the width of 10 fibrils at two sites for each fibril.

MSA, multiple system atrophy; MSA-C, MSA cerebellar variant; MSA-P, MSA Parkinsonian variant; NS, not significant

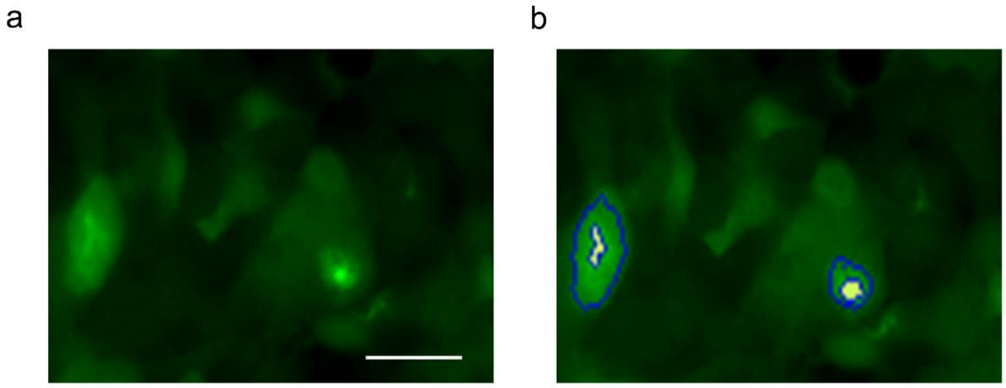

Supplementary Fig. 5. Fluorescence density measurement method

(a) Original image. We confirmed 100 cells per case using the Hybrid Cell Count software.

(b) The fluorescence density of intracellular α-synuclein inclusions generated by seeds derived from patients with Lewy body diseases and MSA was calculated as the fluorescent intensity of inclusion bodies (yellow) divided by the area of inclusions (blue). Hybrid Cell Count software was used to measure the fluorescence intensity. Scale bar: 50 μm.

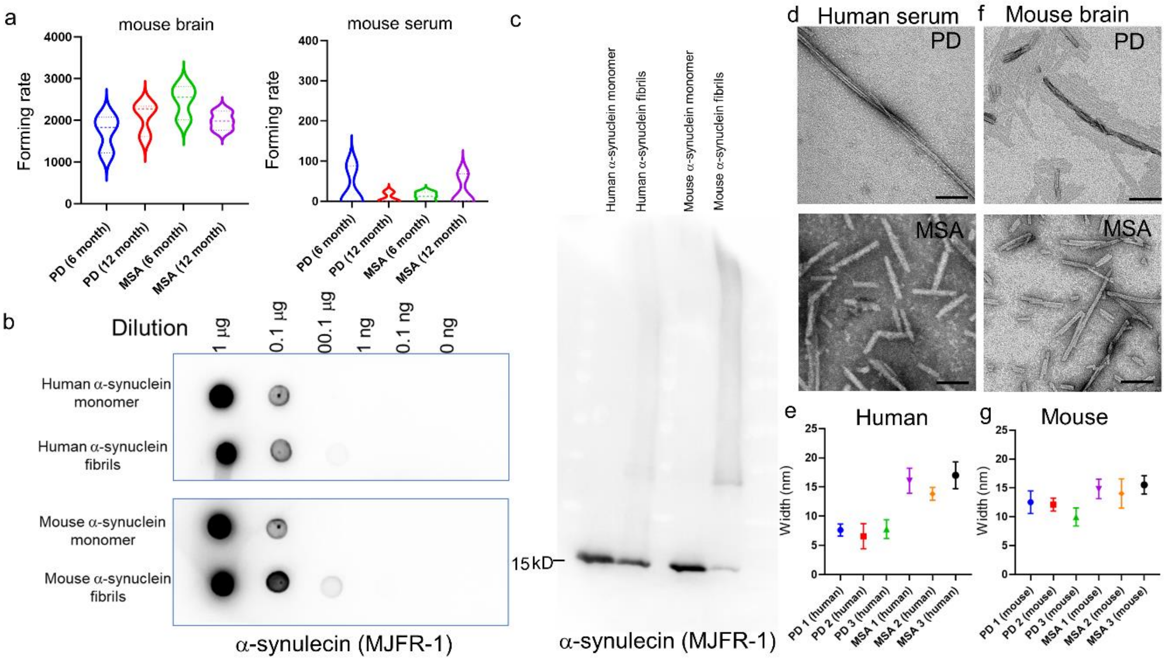

Supplementary Fig. 6. α-synuclein seed properties in the mouse model

(a) Comparison of the forming rates of each group. The violin plots matched those represented in the kinetic curves. Violin plots show the range and average distribution. Dot blot (b) and western blot (repeated two times) (c) confirmed that MJFR-1, an anti-α-synuclein antibody, detected the mouse and human α-synuclein monomer and fibril. Negative-stained transmission electron microscopy images of the original product amplified by human serum IP/RT-QuIC from PD or MSA patients (d, e) and mouse brain RT-QuIC-derived α-synuclein fibrils (f, g) obtained from PD- or MSA-seed injected mouse brain. The widths of α-synuclein fibrils derived from human serum (e) or mouse brain (g) are shown. We measured the width at two sites per fibril (n=3). The data represent mean ± standard error of the mean. Scale bars are 100 nm. MSA, multiple

system atrophy; NS, not significant; PD, Parkinson’s disease.

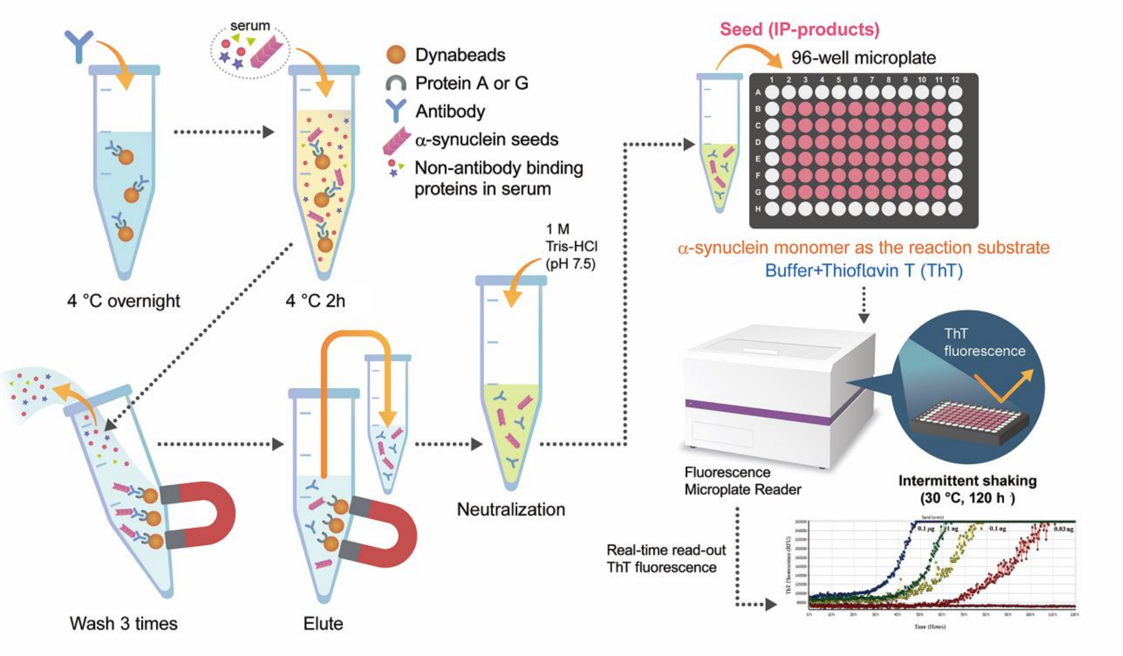

Supplementary Fig. 7. Protocol steps of the IP/RT-QuIC

First, 100 μL of IP lysis buffer (1% BSA, 150 mM NaCl, 1% Triton X, 50 mM Tris HCl, pH 7) containing 1.7 μg MJFR-1 (anti-α-synuclein antibody: Abcam, UK) and 30 μL of protein A/G magnetic beads (Thermo Fisher Scientific, USA) were incubated overnight at 4 °C. Then, 100 μL of serum (1 mg protein/mL) was added to the buffer and rotated at 4 °C for 2 h. The proteins were eluted using 20 μL of 50 mM glycine, and the samples were adjusted to pH 7.5. The reaction buffer (RB) contained 100 mM phosphate buffer (pH 7.5-8.0), 10 μM thioflavin T (ThT), 0-170 mM NaCl, and 0.1 mg/mL recombinant α-synuclein. Each well of a black 96-well plate with a clear bottom (Thermo Fisher Scientific, USA) contained 95 μL of RB and 37 ± 3 mg of 0.5-mm zirconium/silica beads (Thermo Fisher Scientific, USA). Reactions were seeded with 5 μL of IP product solution

from the serum to a final reaction volume of 100 μL. The plates were incubated in a FLUOstar OPTIMA microplate reader (BMG Labtech, Germany) at 30 °C for 120 h with intermittent shaking cycles: double-orbital with 1 min of shaking at 200 rpm followed by 14 min of rest. ThT fluorescence measurements (450 nm excitation and 480 nm emission) were taken every 15 min.

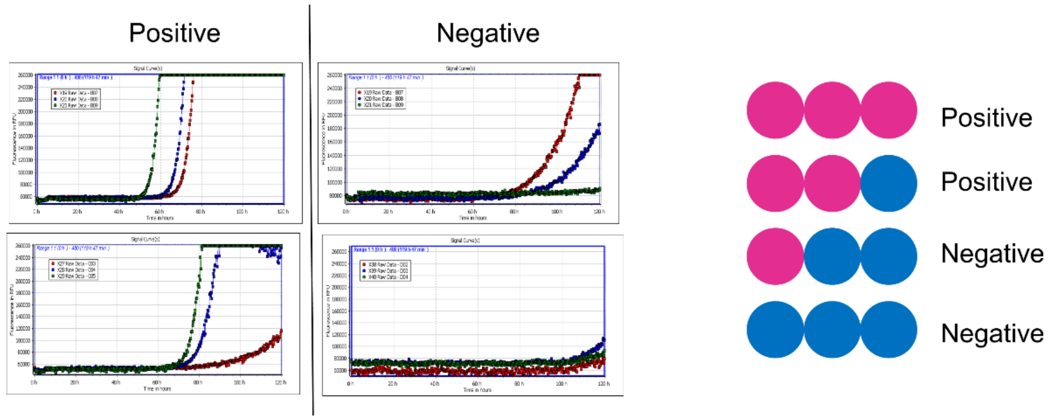

Supplementary Fig. 8. Definition of a positive and negative response in IP/RT-QuIC

A positive response was defined as a relative fluorescence unit value of >260,000 at 120 h. Positive signals in two or more of the triplicate wells were considered positive. It was considered negative if the relative fluorescence unit value did not reach 260,000 within 120 h.

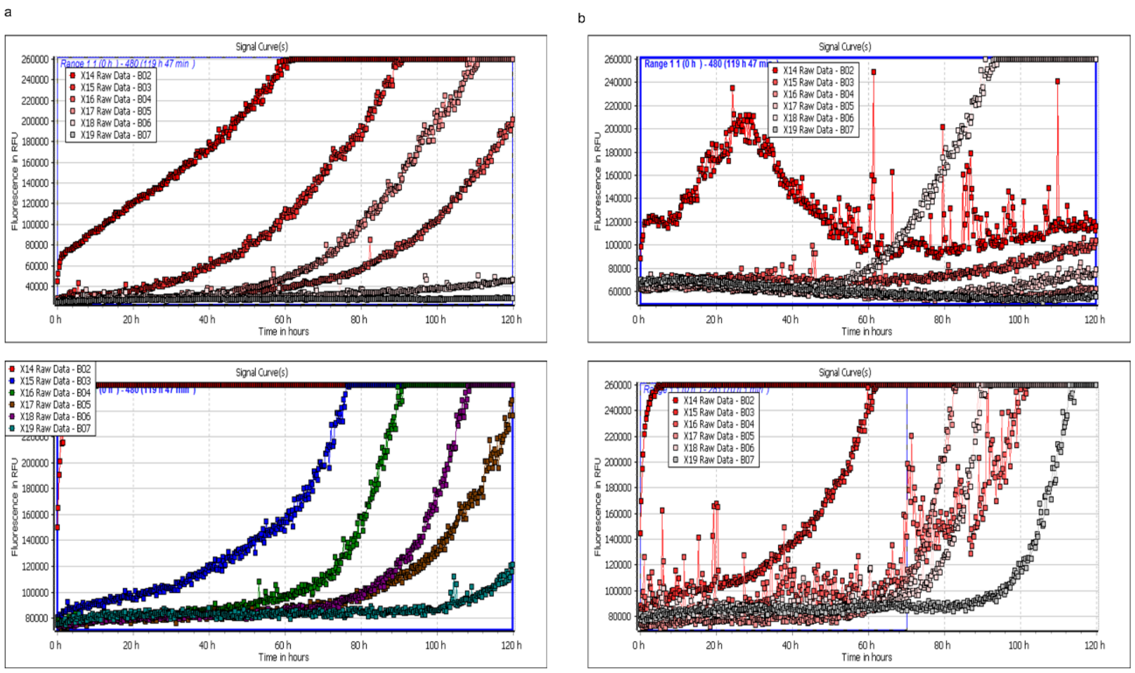

Supplementary Fig. 9. Standard curves of IP/RT-QuIC

We use six different concentrations as standards: 10 ng/µl (B02), 1 ng/µl (B03), 0.1 ng/µl (B04), 0.01 ng/µl (B05), 0.001 ng/µl (B06), and 0 ng/µl (B07). An increase in the fluorescence intensity in the concentration dependence from 10 ng/µl to 0.01 ng/µl ensures that the relative fluorescence unit value reaches 260,000, 0 ng/µl relative fluorescence unit value is less than 260,000, and 0.001 ng/µl may or may not reach 260,000. An increase in the fluorescence intensity indicates that (a) the result of IP/RT-QuIC is reliable. On the contrary, if no increase in the fluorescence intensity of the concentration dependence is observed, (b) the reliability of the results of IP/RT-QuIC at that time is considered low, and all IP/RT-QuIC at that time are re-performed from scratch.

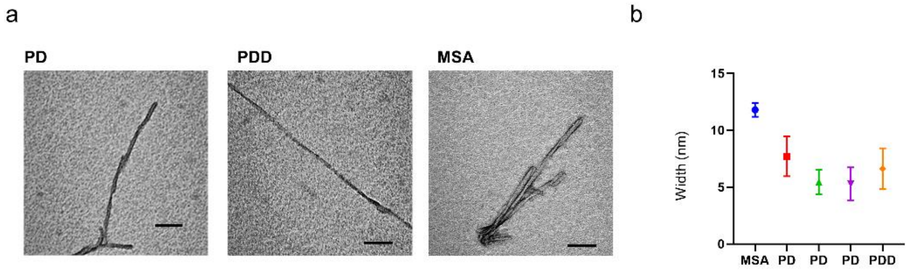

Supplementary Fig. 10. Morphological analysis of amplified products derived from pathologically confirmed synucleinopathies cases with positive IP/RT-QuIC results

(a) Negative-stained transmission electron microscopy images of α-synuclein fibrils derived from pathologically confirmed synucleinopathies. (b) The widths of products derived from PD, PDD, and MSA with positive IP/RT-QuIC results are shown. The data represent mean ± standard error of the mean. We measured the width at two sites per fibril (n=3). Scale bars are 100 nm. MSA, Multiple system atrophy; PD, Parkinson’s disease; PDD, PD with dementia

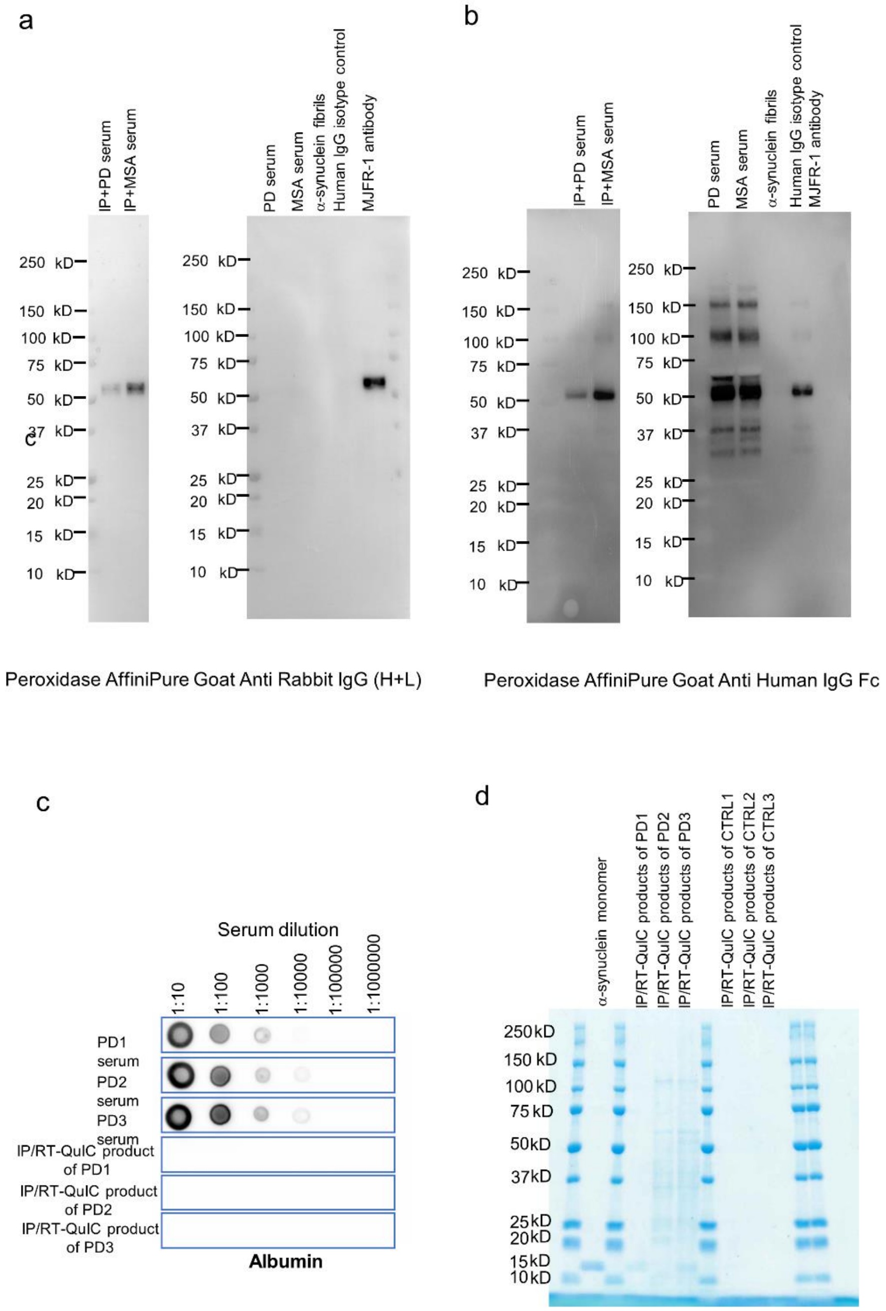

Supplementary Fig. 11. Contaminant proteins in the serum have little effect on aggregation

IP products derived from serum contained human IgG (repeated two times) (a) and antibodies used for IP (repeated two times) (b). Dot blot of serum samples shows albumin

contamination, but IP/RT-QuIC products show that albumin was removed (c). Coomassie brilliant blue assay (CBB) shows that there were not large amounts of contaminants in the IP/RT-QuIC products (repeated two times) (d).

Supplementary Table S1. Percentage distribution of replicates of IP/RT-QuIC

Diagnosis	N	IP/RT-QuIC Results +/-	Number of positive wells (samples, n)			
			Positive		Negative	
Synucleinopathies						
PD	221	210/11	3 (80)	2 (130)	1 (8)	0 (3)
			36%	59%	3.60%	1.40%
MSA	39	25/14	3 (13)	2 (12)	1 (2)	0 (12)
			30.8%	33.3%	5.10%	30.8%
DLB	10	9/1	3 (4)	2 (5)	1 (1)	0 (0)
			40%	50%	10%	0%
Non-synucleinopathies						
PSP	30	1/29	3 (1)	2 (0)	1 (9)	0 (20)
			3%	0%	30%	67%
AD	25	4/21	3 (3)	2 (1)	1 (8)	0 (13)
			12%	4%	32%	52%
Controls	128	11/117	3 (6)	2(5)	1 (44)	0 (73)
			4.7%	3.9%	34.4%	57%
Patients with *PRKN* mutations	17	0/17	3 (0)	2 (0)	1 (4)	0 (13)
			0%	0%	23.5%	76.50%

AD, Alzheimer's disease; DLB, dementia with Lewy bodies; MSA, multiple system atrophy; PD, Parkinson's disease; PSP, progressive supranuclear palsy

Supplementary Table S2. The performance of IP/RT-QuIC in some cases from the first cohort repeated two times and the reproducibility (intra-batch reproducibility: –1)

a

Diagnosis		First result			
		0/3 (n=3)	1/3 (n=5)	2/3 (n=5)	3/3 (n=5)
		Second result (%)	Second result (%)	Second result (%)	Second result (%)
PD	0/3	3 (100)	5 (100)	1 (20)	0 (0)
	1/3	0 (0)	0 (0)	0 (0)	0 (0)
	2/3	0 (0)	0 (0)	0 (0)	0 (0)
	3/3	0 (0)	0 (0)	4 (80)	5 (100)

Diagnosis		First result			
		0/3 (n=5)	1/3 (n=2)	2/3 (n=5)	3/3 (n=5)
		Second result (%)	Second result (%)	Second result (%)	Second result (%)
MSA	0/3	4 (80)	1 (50)	0 (0)	0
	1/3	1 (20)	1 (50)	0 (0)	0
	2/3	0 (0)	0 (0)	3 (60)	1 (20)
	3/3	0 (0)	0 (0)	2 (40)	4 (80)

Diagnosis		First result			
		0/3 (n=10)	1/3 (n=25)	2/3 (n=5)	3/3 (n=6)
		Second result (%)	Second result (%)	Second result (%)	Second result (%)

CTRL	0/3	7 (70)	13 (52)	1 (20)	0 (0)
	1/3	2 (20)	11(44)	0 (0)	0 (0)
	2/3	0 (0)	0 (0)	3 (60)	2 (33.3)
	3/3	1 (10)	1 (4)	1 (20)	4 (66.7)

b

Simple kappa coefficient

Pair	Estimate	SE	95% CI
First-second			
PD (n=18)	0.8900	0.107	0.68-1
MSA (n=17)	1.0000	0	1-1
CTRL (n=46)	0.8300	0.097	0.64-1

CTRL, control; MSA, multiple system atrophy; PD, Parkinson’s disease; SE, standard error; CI, confidence interval

Supplementary Table S3. IP/RT-QuIC positive/negative reproducibility was analyzed using samples collected at different dates and times from the same cases (intra-batch reproducibility: –2)

Diagnosis	Day 1		Day 2	
	Positive wells	Judgment	Positive wells	Judgment
PD	3/3	Positive	3/3	Positive
PD	3/3	Positive	2/3	Positive
PD	3/3	Positive	3/3	Positive
MSA	3/3	Positive	3/3	Positive
MSA	3/3	Positive	3/3	Positive
MSA	3/3	Positive	3/3	Positive
DLB	2/3	Positive	3/3	Positive
DLB	3/3	Positive	3/3	Positive
DLB	3/3	Positive	3/3	Positive
CTRL	0/3	Negative	1/3	Negative
CTRL	0/3	Negative	0/3	Negative
CTRL	0/3	Negative	0/3	Negative

Judgment

Simple kappa

coefficient

Pair	Estimate	SE	95% CI
Total, n=12			
Day 1-day 2	1.000		1.000 -1.000

Well-positive

Simple kappa

coefficient

Pair	Estimate	SE	95% CI
Total, n=12			
Day 1-day 2	0.710		0.1084-0.9053

CTRL, control; DLB, dementia with Lewy bodies; MSA, multiple system atrophy; PD, Parkinson’s disease; SE, standard error; CI, confidence interval

Supplementary Table S4. Reproducibility for result determination by three independent examiners (inter-batch reproducibility: –1)

Diagnosis	Evaluator A		Evaluator B		Evaluator C	
	Positive wells	Judgement	Positive wells	Judgement	Positive wells	Judgement
PD	1/3	Negative	1/3	Negative	1/3	Negative
PD	1/3	Negative	1/3	Negative	1/3	Negative
PD	3/3	Positive	3/3	Positive	3/3	Positive
PD	2/3	Positive	2/3	Positive	2/3	Positive
PD	2/3	Positive	2/3	Positive	2/3	Positive
PD	2/3	Positive	2/3	Positive	2/3	Positive
PD	3/3	Positive	3/3	Positive	3/3	Positive
PD	3/3	Positive	3/3	Positive	3/3	Positive
PD	2/3	Positive	2/3	Positive	2/3	Positive
PD	3/3	Positive	1/3	Negative	2/3	Positive
PD	3/3	Positive	3/3	Positive	3/3	Positive
PD	2/3	Positive	2/3	Positive	2/3	Positive
PD	3/3	Positive	3/3	Positive	3/3	Positive
PD	3/3	Positive	3/3	Positive	3/3	Positive
PD	3/3	Positive	3/3	Positive	3/3	Positive
PD	2/3	Positive	1/3	Negative	1/3	Negative
PD	1/3	Negative	1/3	Negative	1/3	Negative
PD	2/3	Positive	1/3	Negative	1/3	Negative
PD	0/3	Negative	0/3	Negative	0/3	Negative
PD	3/3	Positive	3/3	Positive	3/3	Positive
PD	3/3	Positive	3/3	Positive	3/3	Positive
PD	1/3	Negative	1/3	Negative	1/3	Negative
PD	0/3	Negative	0/3	Negative	0/3	Negative
PD	0/3	Negative	0/3	Negative	0/3	Negative
PD	2/3	Positive	1/3	Negative	1/3	Negative
MSA	3/3	Positive	3/3	Positive	3/3	Positive
MSA	3/3	Positive	3/3	Positive	3/3	Positive
MSA	1/3	Negative	1/3	Negative	1/3	Negative
MSA	2/3	Positive	2/3	Positive	2/3	Positive
MSA	3/3	Positive	3/3	Positive	3/3	Positive

MSA	3/3	Positive	3/3	Positive	3/3	Positive
MSA	0/3	Negative	1/3	Negative	0/3	Negative
MSA	2/3	Positive	1/3	Negative	1/3	Negative
MSA	1/3	Negative	1/3	Negative	1/3	Negative
MSA	3/3	Positive	3/3	Positive	3/3	Positive
MSA	2/3	Positive	1/3	Negative	1/3	Negative
MSA	2/3	Positive	1/3	Negative	1/3	Negative
MSA	0/3	Negative	0/3	Negative	0/3	Negative
MSA	2/3	Positive	2/3	Positive	2/3	Positive
MSA	2/3	Positive	2/3	Positive	2/3	Positive
MSA	0/3	Negative	0/3	Negative	0/3	Negative
DLB	3/3	Positive	3/3	Positive	3/3	Positive
DLB	3/3	Positive	3/3	Positive	3/3	Positive
DLB	1/3	Negative	1/3	Negative	1/3	Negative
DLB	2/3	Positive	2/3	Positive	2/3	Positive
DLB	2/3	Positive	2/3	Positive	2/3	Positive
CTRL	1/3	Negative	1/3	Negative	1/3	Negative
CTRL	1/3	Negative	1/3	Negative	1/3	Negative
CTRL	0/2	Negative	0/2	Negative	0/2	Negative
CTRL	0/2	Negative	0/2	Negative	0/2	Negative
CTRL	0/2	Negative	0/2	Negative	0/2	Negative
CTRL	0/2	Negative	0/2	Negative	0/2	Negative
CTRL	0/2	Negative	0/2	Negative	0/2	Negative
CTRL	0/2	Negative	0/2	Negative	0/2	Negative
CTRL	0/2	Negative	0/2	Negative	0/2	Negative
CTRL	0/2	Negative	0/2	Negative	0/2	Negative
CTRL	0/2	Negative	0/2	Negative	0/2	Negative
CTRL	0/2	Negative	0/2	Negative	0/2	Negative
CTRL	0/2	Negative	0/2	Negative	0/2	Negative
CTRL	0/2	Negative	0/2	Negative	0/2	Negative
CTRL	0/2	Negative	0/2	Negative	0/2	Negative
CTRL	0/2	Negative	0/2	Negative	0/2	Negative
CTRL	0/2	Negative	0/2	Negative	0/2	Negative
CTRL	0/3	Negative	0/3	Negative	0/3	Negative
CTRL	3/3	Positive	3/3	Positive	3/3	Positive
CTRL	0/3	Negative	0/3	Negative	0/3	Negative

CTRL	1/3	Negative	1/3	Negative	1/3	Negative
CTRL	1/3	Negative	1/3	Negative	1/3	Negative
CTRL	1/3	Negative	1/3	Negative	1/3	Negative
CTRL	0/3	Negative	0/3	Negative	0/3	Negative

Judgement

Simple kappa coefficient

Pair	Estimate	SE	95% CI
Total, n=70			
A-B	0.7987	0.0707	0.6601-0.9373
A-C	0.8276	0.0663	0.6976-0.9575
B-C	0.9701	0.0297	0.9118-1.0000

Well-positive

Judge

Simple kappa coefficient

Pair	Estimate	SE	95% CI
Total, n=70			
A-B	0.8462	0.0498	0.7487-0.9438
A-C	0.8649	0.0473	0.7722-0.9575
B-C	0.961	0.0272	0.9078-1.0000

CTRL, control; DLB, dementia with Lewy bodies; MSA, multiple system atrophy; PD, Parkinson’s disease; SE, standard error; CI, confidence interval

Supplementary Table S5. Reproducibility of the IP/RT-QuIC technique assessed by two independent examiners (inter-batch reproducibility: –2)

Diagnosis	Examiner A		Examiner B	
	Positive Wells	Judgement	Positive Wells	Judgement
PD	2/3	Positive	3/3	Positive
PD	2/3	Positive	3/3	Positive
PD	2/3	Positive	3/3	Positive
PD	2/3	Positive	3/3	Positive
PD	3/3	Positive	2/3	Positive
PD	3/3	Positive	3/3	Positive
PD	3/3	Positive	2/3	Positive
PD	2/3	Positive	2/3	Positive
PD	2/3	Positive	3/3	Positive
PD	3/3	Positive	3/3	Positive
MSA	2/3	Positive	3/3	Positive
MSA	1/3	Negative	3/3	Positive
MSA	3/3	Positive	3/3	Positive
MSA	1/3	Negative	2/3	Positive
MSA	1/3	Negative	2/3	Positive
MSA	2/3	Positive	3/3	Positive
MSA	1/3	Negative	1/3	Negative
MSA	3/3	Positive	3/3	Positive
MSA	3/3	Positive	3/3	Positive
MSA	3/3	Positive	3/3	Positive
DLB	2/3	Positive	3/3	Positive
DLB	3/3	Positive	0/3	Negative
DLB	3/3	Positive	2/3	Positive
DLB	3/3	Positive	2/3	Positive
DLB	3/3	Positive	3/3	Positive
DLB	3/3	Positive	3/3	Positive
DLB	2/3	Positive	0/3	Negative
DLB	3/3	Positive	3/3	Positive
DLB	2/3	Positive	1/3	Negative
DLB	3/3	Positive	3/3	Positive

CTRL	0/3	Negative	1/3	Negative
CTRL	0/3	Negative	0/3	Negative
CTRL	0/3	Negative	0/3	Negative
CTRL	1/3	Negative	0/3	Negative
CTRL	0/3	Negative	0/3	Negative
CTRL	0/3	Negative	3/3	Positive

Simple kappa coefficient

Pair	Estimate	SE	95% CI
Total, n=36			
	0.680	0.150	0.390-0.970

CTRL, control; DLB, dementia with Lewy bodies; MSA, multiple system atrophy; PD, Parkinson's disease; SE, standard error; CI, confidence interval

Supplementary Table S6. Serum α-synuclein IP/RT-QuIC results of MSA-P and MSA-C cases.

Diagnosis	N	IP/RT-QuIC Results +/-	Positive results
MSA	39	25/14	64%
MSA-P	26	17/9	65%
MSA-C	13	8/5	62%

Data are presented as numbers. IP/RT-QuIC, immunoprecipitation/real-time quaking-induced conversion; MSA, multiple system atrophy; MSA-C, MSA cerebellar variant; MSA-P, MSA Parkinsonian variant; N, number of participants who received IP/RT-QuIC

Supplementary Table S7. The average age of the control cases with positive IP/RT-QuIC results.

Age (years)	N (%)
20–30	0/2 (0)
31–40	0/5 (0)
41–50	1/28 (3.6)
51–60	0/17 (0)
61–70	1/21 (4.7)
71–80	5/38 (10)
81–90	4/17 (23)

Data are presented as numbers (%). N, number of participants who received IP/RT-QuIC

Supplementary Table S8. The correlation between IP/RT-QuIC parameters and the MIBG H/M ratio of (a) PD and (B) MSA cases.

a

PD	MIBG H/M ratio reduced	MIBG H/M ratio normal	p-value
IP/RT-QuIC-positive	127	19	
IP/RT-QuIC-negative	0	2	0.0193
Total	127	21	

b

MSA	MIBG H/M ratio reduced	MIBG H/M ratio normal	p-value
IP/RT-QuIC-positive	2	9	
IP/RT-QuIC-negative	1	7	NS
Total	3	16	

Data are presented as numbers. P-values were obtained using (a) Pearson’s chi-squared test and (b) Fisher’s exact test. IP/RT-QuIC, immunoprecipitation/real-time quaking-induced conversion; MIBG, ^{123}I-metaiodobenzylguanidine scintigraphy; MSA, multiple system atrophy; PD, Parkinson’s disease

Supplementary Table S9. The correlation between IP/RT-QuIC results and synucleinopathies (PD and MSA).

	**IP/RT-QuIC-positive**	**IP/RT-QuIC-negative**	**p-value**
PD	210	11	
MSA	25	14	<0.0001
Total	235	25	

Data are presented as numbers. P-values were obtained using Pearson's chi-square test (p<0.0001).

IP/RT-QuIC, immunoprecipitation/real-time quaking-induced conversion; MSA, multiple system atrophy; PD, Parkinson's disease

Supplementary Table S10. Characteristics of the study participants from whom both serum and CSF samples were obtained

	**Controls (n=35)**	**Parkinson's disease (n=6)**	**Multiple system atrophy (n=3)**
Age [years], mean (SD)	52 (18)	63 (9.2)	61 (16)
Men, n (%)	22 (63)	2 (33)	1 (33)
Hoehn–Yahr stages, mean (SD)	NA	2.8 (0.9)	3.0 (0.8)
UPDRS-III, mean (SD)	NA	29 (9.2)	36 (14)
Disease duration [years], mean (SD)	NA	9.5 (3.0)	4.6 (1.2)

Both serum and CSF data are available from 9 patients (6 PD and 3 MSA) and 35 controls.

CSF, cerebrospinal fluid; MSA, multiple system atrophy; NA, Not Applicable; SD, standard deviation; UPDRS-III, Unified Parkinson's Disease Rating Scale Part III

Supplementary Table S11. Reproducibility for cell assay determination by two independent examiners (intra-batch/inter-batch reproducibility)

Diagnosis	Judgement
PD	Correct
PD	Correct
PD	Correct
PD	Correct
PD	Correct
PD	Correct
PD	Correct
PD	Correct
PD	Correct
PD	Correct
PD	Correct
PD	Correct
PD	Correct
PD	Correct
PD	Correct
PD	Correct
PD	Correct
PD	Correct
PD	Correct
PD	Correct
MSA	Correct
MSA	Correct
MSA	Correct
MSA	Correct
MSA	Correct
MSA	Correct
MSA	Correct
MSA	Correct
MSA	Failure
MSA	Failure
MSA	Correct
MSA	Correct

MSA	Failure
MSA	Failure
MSA	Failure
MSA	Failure
MSA	Correct
MSA	Correct
MSA	Correct
MSA	Correct
DLB	Correct
DLB	Failure
DLB	Correct
DLB	Failure
DLB	Correct
Accuracy rate	
PD	100%
MSA	70%
DLB	60%

Intra-batch

Diagnosis	Judge1	Judgement	Judge 2	Judgement
PD	PD	Correct	PD	Correct
PD	PD	Correct	PD	Correct
PD	PD	Correct	PD	Correct
MSA	MSA	Correct	MSA	Correct
MSA	DLB	Failure	MSA	Correct
MSA	MSA	Correct	MSA	Correct
DLB	DLB	Correct	DLB	Correct
DLB	DLB	Correct	MSA	Failure
DLB	DLB	Correct	DLB	Correct
CTRL	CTRL	Correct	CTRL	Correct
CTRL	CTRL	Correct	CTRL	Correct

Simple kappa coefficient

Pair	Estimate	SE	95% CI

Total, n=11			
	0.84	0.103	0.64-1

Inter-batch

Diagnosis	Evaluator A	Evaluator B
PD	PD	PD
PD	PD	PD
MSA	MSA	DLB
MSA	MSA	MSA
DLB	DLB	DLB
DLB	DLB	MSA
CTRL	CTRL	CTRL

Simple kappa coefficient

Pair	Estimate	SE	95% CI
Total, n=7			
	0.75	0.177	0.404-1

Diagnosis	Evaluator B	Evaluator C
PD	PD	PD
PD	PD	PD
PD	PD	PD
PD	PD	PD
PD	PD	PD
PD	PD	PD
PD	PD	PD
PD	PD	PD
PD	PD	PD
PD	PD	PD
MSA	DLB	DLB

MSA	MSA	MSA
MSA	MSA	MSA
MSA	MSA	MSA
MSA	MSA	MSA
MSA	MSA	MSA
MSA	DLB	DLB
DLB	DLB	DLB
DLB	DLB	DLB
DLB	DLB	DLB
DLB	DLB	DLB
DLB	DLB	DLB
DLB	DLB	DLB

Simple kappa coefficient

Pair	Estimate	SE	95% CI
Total, n=23			
	1.00	0	1-1

Evaluator A

Diagnosis	Predicted pathology	FL type	DC type	PC type	Not determined	Highest%
PD	PD	90.3%	3.2%	0.0%	6.5%	90.3%
PD	PD	91.7%	8.3%	0.0%	0.0%	91.7%
PD	PD	100.0%	0.0%	0.0%	0.0%	100.0%
PD	PD	94.1%	2.9%	0.0%	2.9%	94.1%
PD	PD	92.9%	7.1%	0.0%	0.0%	92.9%
PD	PD	91.7%	8.3%	0.0%	0.0%	91.7%
MSA	MSA	0.0%	80.0%	20.0%	0.0%	80.0%
MSA	DLB/PDD	0.0%	18.2%	81.8%	0.0%	81.8%
MSA	DLB/PDD	7.1%	14.3%	78.6%	0.0%	78.6%
MSA	MSA	0.0%	81.3%	18.8%	0.0%	81.3%
MSA	MSA	0.0%	78.6%	21.4%	0.0%	78.6%
DLB/PDD	DLB/PDD	0.0%	0.0%	100.0%	0.0%	100.0%
DLB/PDD	DLB/PDD	0.0%	0.0%	85.7%	14.3%	85.7%

DLB/PDD	DLB/PDD	0.0%	14.3%	57.1%	28.6%	57.1%
DLB/PDD	DLB/PDD	0.0%	37.5%	50.0%	12.5%	50.0%
DLB/PDD	DLB/PDD	0.0%	0.0%	100.0%	0.0%	100.0%

Evaluator B

Diagnosis	Predicted pathology	FL type	DC type	PC type	Not determined	Highest%
PD	PD	90.6%	0.0%	3.1%	6.3%	90.6%
PD	PD	86.4%	0.0%	4.5%	9.1%	86.4%
PD	PD	76.5%	5.9%	5.9%	11.8%	76.5%
PD	PD	91.9%	0.0%	5.4%	2.7%	91.9%
PD	PD	72.7%	9.1%	0.0%	18.2%	72.7%
PD	PD	85.7%	0.0%	0.0%	14.3%	85.7%
MSA	MSA	0.0%	82.4%	11.8%	5.9%	82.4%
MSA	DLB/PDD	12.5%	33.3%	50.0%	4.2%	50.0%
MSA	DLB/PDD	5.0%	40.0%	50.0%	5.0%	50.0%
MSA	MSA	16.7%	55.6%	22.2%	5.6%	55.6%
MSA	MSA	5.6%	66.7%	22.2%	5.6%	66.7%
DLB/PDD	DLB/PDD	0.0%	0.0%	84.6%	15.4%	84.6%
DLB/PDD	DLB/PDD	9.1%	9.1%	81.8%	0.0%	81.8%
DLB/PDD	DLB/PDD	6.7%	26.7%	53.3%	13.3%	53.3%
DLB/PDD	DLB/PDD	18.2%	27.3%	45.5%	9.1%	45.5%
DLB/PDD	DLB/PDD	0.0%	18.2%	72.7%	9.1%	72.7%

Evaluator C

Diagnosis	Predicted pathology	FL type	DC type	PC type	Not determined	Highest%
PD	PD	85.7%	2.9%	8.6%	2.9%	85.7%
PD	PD	84.0%	0.0%	12.0%	4.0%	84.0%
PD	PD	85.7%	0.0%	9.5%	4.8%	85.7%
PD	PD	94.6%	0.0%	0.0%	5.4%	94.6%
PD	PD	87.5%	4.2%	4.2%	4.2%	88.0%
PD	PD	80.0%	5.0%	15.0%	0.0%	80.0%
MSA	MSA	30.0%	40.0%	25.0%	5.0%	40.0%
MSA	MSA	10.0%	45.0%	40.0%	5.0%	45.0%
MSA	MSA	17.4%	47.8%	34.8%	0.0%	47.8%

MSA	MSA	5.3%	47.4%	36.8%	10.5%	47.0%
MSA	MSA	10.5%	52.6%	31.6%	5.3%	52.6%
DLB/PDD	DLB/PDD	0.0%	0.0%	92.9%	7.1%	92.9%
DLB/PDD	DLB/PDD	0.0%	30.0%	70.0%	0.0%	70.0%
DLB/PDD	DLB/PDD	8.3%	25.0%	58.3%	8.3%	58.3%
DLB/PDD	DLB/PDD	10.0%	10.0%	80.0%	0.0%	80.0%
DLB/PDD	DLB/PDD	0.0%	16.7%	66.7%	16.7%	67.0%

CTRL, control; DLB, dementia with Lewy bodies; MSA, multiple system atrophy; PD, Parkinson’s disease; PDD, PD with dementia; SE, standard error

Supplementary Table S12. Results of the IP/RT-QuIC of the mouse brain (a) and serum (b) after the injection of PD- or MSA-seeds into the mouse brain

a

		Positive wells	PD-seed-injected brain (6 months) n=3	PD-seed-injected brain (1 year) n=3	MSA-seed-injected brain (6 months) n=3	MSA-seed-injected brain (1 year) n=3
RT-QuIC	Negative	0/3 (%)	0 (0)	0 (0)	0 (0)	0 (0)
		1/3 (%)	0 (0)	0 (0)	0 (0)	0 (0)
	Positive	2/3 (%)	0 (0)	0 (0)	0 (0)	0 (0)
		3/3 (%)	3 (100)	3 (100)	3 (100)	3 (100)

b

		Positive wells	PD-seed-injected mouse serum (6 months) n=3	PD-seed-injected mouse serum (1 year) n=3	MSA-seed-injected mouse serum (6 months) n=3	MSA-seed-injected mouse serum (1 year) n=3
IP/RT-QuIC	Negative	0/3 (%)	3 (100)	2 (67)	3 (100)	3 (100)
		1/3 (%)	0 (0)	1 (33)	0 (0)	0 (0)
	Positive	2/3 (%)	0 (0)	0 (0)	0 (0)	0 (0)
		3/3 (%)	0 (0)	0 (0)	0 (0)	0 (0)

IP, Immunoprecipitation; MSA, multiple system atrophy; PD, Parkinson’s disease; RT-QuIC, real-time quaking-induced conversion

Supplementary Table S13. The correlation between IP/RT-QuIC parameters and characteristics and clinical parameters of rapid eye movement sleep behavior disorder.

	Forming Rate		$T_{1/2}$		T_{max}		AUC	
	**R**	***P***	**r**	***p***	**r**	***p***	**r**	***p***
Age	0.4136	0.1814	0.2781	0.3814	0.2888	0.3627	0.2992	0.3448
UPDRS-III	-0.04914	0.8794	-0.114	0.7242	-0.1123	0.7282	-0.08874	0.7839
Disease Duration	0.7315	0.0069	0.5158	0.0861	0.5267	0.0785	0.7685	0.0035
SBR	-0.8331	0.0053	-0.803	0.0092	-0.8042	0.009	-0.7396	0.0227

Correlations between variables were assessed using *two-tailed p*earson correlation analyses.

Underlined values indicate p<0.05. AUC, area under the curve; SBR, specific binding ratio; $T_{1/2}$, time to reach 130k fluorescence; T_{max}, time to reach maximum (260k) fluorescence; UPDRS-III, Unified Parkinson's Disease Rating Scale Part III

Supplementary Table S14. Mutations in *Parkin*-linked Parkinson's disease cases.

Exon 3 deletion (heterozygous)/c.535-3A>G (p.G179RfsX10) (heterozygous)
Exon 2-4 del/exon 4 del (compound heterozygous)
Exon 3 deletion (heterozygous)/exon 4 deletion (heterozygous)
Exon 4 deletion (homozygous)
Exon 2-4 deletion (heterozygous)/c.535-3A>G (p.G179RfsX10) (heterozygous)
Exon 6-7 deletion (homozygous)
Exon 2-4 deletion (homozygous)
Exon 2-3 deletion (heterozygous)/c.1358G>A (p.W453X) (heterozygous)
Exon 5 deletion (homozygous)
Exon 3-5 deletion (heterozygous)/exon 3-7 duplication (heterozygous)
Exon 6 duplication (homozygous)
Exon 2-4 deletion (heterozygous)/exon 5-6 deletion (heterozygous)
Exon 2-4 deletion (homozygous)
c.818G>A (p.N273S) (heterozygous)
c.535delG (p.G179VfsX9) (heterozygous)
c.1358G>A (p.W453X) (heterozygous)

Exon 3 deletion (homozygous)
