# Supplementary material for: Propagative α-synuclein seeds as serum biomarkers for synucleinopathies
Source: Nat Med. 2023 May 29;29(6):1448–55. doi: 10.1038/s41591-023-02358-9 (PMC10287557; doi:10.1038/s41591-023-02358-9)
Supplement: Supplementary file 2 — Reporting Summary [file 41591_2023_2358_MOESM2_ESM.pdf]

nature portfolio

Corresponding author(s): Nobutaka Hattori

Last updated by author(s): Apr 17, 2023

# Reporting Summary

Nature Portfolio wishes to improve the reproducibility of the work that we publish. This form provides structure for consistency and transparency in reporting. For further information on Nature Portfolio policies, see our Editorial Policies and the Editorial Policy Checklist.

## Statistics

For all statistical analyses, confirm that the following items are present in the figure legend, table legend, main text, or Methods section.

| n/a | Confirmed | |
|---|---|---|
| ☐ | ☒ | The exact sample size (*n*) for each experimental group/condition, given as a discrete number and unit of measurement |
| ☐ | ☒ | A statement on whether measurements were taken from distinct samples or whether the same sample was measured repeatedly |
| ☐ | ☒ | The statistical test(s) used AND whether they are one- or two-sided<br>*Only common tests should be described solely by name; describe more complex techniques in the Methods section.* |
| ☒ | ☐ | A description of all covariates tested |
| ☐ | ☒ | A description of any assumptions or corrections, such as tests of normality and adjustment for multiple comparisons |
| ☒ | ☐ | A full description of the statistical parameters including central tendency (e.g. means) or other basic estimates (e.g. regression coefficient) AND variation (e.g. standard deviation) or associated estimates of uncertainty (e.g. confidence intervals) |
| ☐ | ☒ | For null hypothesis testing, the test statistic (e.g. *F*, *t*, *r*) with confidence intervals, effect sizes, degrees of freedom and *P* value noted<br>*Give P values as exact values whenever suitable.* |
| ☒ | ☐ | For Bayesian analysis, information on the choice of priors and Markov chain Monte Carlo settings |
| ☒ | ☐ | For hierarchical and complex designs, identification of the appropriate level for tests and full reporting of outcomes |
| ☒ | ☐ | Estimates of effect sizes (e.g. Cohen's *d*, Pearson's *r*), indicating how they were calculated |

*Our web collection on statistics for biologists contains articles on many of the points above.*

## Software and code

Policy information about availability of computer code

| | |
|---|---|
| Data collection | See below for full description of collection sofrware.<br>IP/RT-QuIC: FLUOstar OPTIMA microplate reader (BMG Labtech, Germany)<br>Imaging and immunohistochemistry: Super-resolution structured illumination microscopy (SR-SIM, Zeiss ELYRA), BZ- X800 (Keyence, Japan)<br>Electron microscopy: HT7700 TEM (Hitachi, Japan) |
| Data analysis | The density of α-synuclein aggregates was quantified using the Hybrid Cell Count software (BZ- X800 1.1.1.8, Keyence, Japan). IBM SPSS (version 22.0; IBM Corp., Armonk, NY, USA), SAS version 9.4 (SAS Institute Inc., Cary, NC, USA), and GraphPad Prism version 8.0 (GraphPad Software, Inc., San Diego, CA, USA) were used for data analysis. |

For manuscripts utilizing custom algorithms or software that are central to the research but not yet described in published literature, software must be made available to editors and reviewers. We strongly encourage code deposition in a community repository (e.g. GitHub). See the Nature Portfolio guidelines for submitting code & software for further information.

## Data

Policy information about availability of data

All manuscripts must include a data availability statement. This statement should provide the following information, where applicable:

- Accession codes, unique identifiers, or web links for publicly available datasets
- A description of any restrictions on data availability
- For clinical datasets or third party data, please ensure that the statement adheres to our policy

Data Availability:The authors declare that all relevant data used to conduct the analyses are available within the article. To protect the privacy and confidentiality of patients in this study, clinical data are not made publicly available in a repository or the supplementary material of the article but can be requested at any time from the corresponding author. Any requests will be reviewed within a time frame of 2 to 3months by the Ethics Committee of Juntendo University to verify whether the request is subject to any intellectual property or confidentiality obligations. All data shared will be de-identified.

## Human research participants

Policy information about studies involving human research participants and Sex and Gender in Research.

| | |
|---|---|
| Reporting on sex and gender | In this study, sex and/or gender data was determined based on biological characteristics from our medical records. |
| Population characteristics | Population characteristics are listed in Table 1 and Extended Data Table 1. The diagnosis was based on standard criteria ( Supplementary Methods). |
| Recruitment | Participating investigators at Juntendo University and the University of Luxembourg recruited participants. The Investigator at each center ensured that the patients were given complete and adequate oral and written information for the study. Participants with neurodegenerative disorders were diagnosed based on the clinical criteria. |
| Ethics oversight | This study was approved by the Ethics Committee of Juntendo University (No. 2021100) and National Ethics Board (CNER Ref: 201407/13) and Data Protection Committee (CNPD Ref: 446/2017) of University of Luxembourg. Written informed consent was obtained from all the patients prior to enrollment. |

Note that full information on the approval of the study protocol must also be provided in the manuscript.

# Field-specific reporting

Please select the one below that is the best fit for your research. If you are not sure, read the appropriate sections before making your selection.

☒ Life sciences ☐ Behavioural & social sciences ☐ Ecological, evolutionary & environmental sciences

For a reference copy of the document with all sections, see nature.com/documents/nr-reporting-summary-flat.pdf

# Life sciences study design

All studies must disclose on these points even when the disclosure is negative.

| | |
|---|---|
| Sample size | We expected that the lower limit for the 97% CI of sensitivity was satisfied in 95% of patients for an estimated sample size of 300 subjects (n=235). Based on the expectation, we determined the available sample size by the number of patients with evaluable blood samples. |
| Data exclusions | No data were excluded from the analyses. |
| Replication | Results were replicated in independent experiments as described in the supplementary data and figure legends. We also conducted second cohort, external cohort and internal/external cohort with pathologically confirmed cases for IP/RT-QuIC. All experiments to replicate the results were successful. |
| Randomization | The study is observational. Therefore, randomization was not needed. |
| Blinding | The experiments from two inner cohorts were an open-label non-blinding study, but we confirmed reproducibility. Furthermore, we performed a blinding survey from an external cohort and confirmed similar results. Morphological analyses using TEM and cell based assay, and analysis of density of intracellular inclusions using cells were blinded. |

# Reporting for specific materials, systems and methods

We require information from authors about some types of materials, experimental systems and methods used in many studies. Here, indicate whether each material, system or method listed is relevant to your study. If you are not sure if a list item applies to your research, read the appropriate section before selecting a response.

## Materials & experimental systems

| n/a | Involved in the study |
| --- | --- |
| ☐ | ☒ Antibodies |
| ☐ | ☒ Eukaryotic cell lines |
| ☒ | ☐ Palaeontology and archaeology |
| ☐ | ☒ Animals and other organisms |
| ☒ | ☐ Clinical data |
| ☒ | ☐ Dual use research of concern |

## Methods

| n/a | Involved in the study |
| --- | --- |
| ☒ | ☐ ChIP-seq |
| ☒ | ☐ Flow cytometry |
| ☒ | ☐ MRI-based neuroimaging |

# Antibodies

Antibodies used

Primary antibodies: anti-α-synuclein antibody (rabbit monoclonal, MJFR1, 1.7 μg for immunoprecipitationim, 1:1000 for Immunoblotting and Dot blot, ab138501, Abcam UK), anti-p-syn antibody (mouse monoclonal, pSyn #64, 1:300, Wako-Fujifilm Japan), neuronal marker (rabbit polyclonal, Anti-NeuN, 1:500, ab104225, Abcam UK), oligodendrocyte marker (rabbit polyclonal, Anti-GST-pi, 1:500, 312, MBL Japan), anti albumin antibody (mouse monoclonal, 1:10000, PGI 4A1C11 Japan), 2) secondary antibodies: biotinylated anti rabbit IgG (1:300, BA1000, Vector), biotinylated anti mouse IgG (1:300, BA9200, Vector), Peroxidase affinipure goat anti rabbit Ig G (1:10000, 111-035-144, Jackson ImmunoResearch USA), Goat anti human IgG (HRP) preadsorbed ( 1:10000, ab98624, Abcam UK)

Validation

Commercial primary antibodies were validated by the manufacturer (See the links)
Anti-α-synuclein antibody (MJFR1): https://www.abcam.co.jp/products/primary-antibodies/alpha-synuclein-antibody-mjfr1-ab138501.html
Anti-p-syn antibody (pSyn #64): https://labchem-wako.fujifilm.com/jp/product/detail/W01W0101-2519.html
Oligodendrocyte marker (Anti-GST-pi): https://ruo.mbl.co.jp/bio/dtl/A/?pcd=312
Anti albumin antibody:https://search.cosmobio.co.jp/view/p_view.asp?PrimaryKeyValue=5371298&ServerKey=&selPrice=1

# Eukaryotic cell lines

Policy information about cell lines and Sex and Gender in Research

Cell line source(s)

HEK293 cell a laboratory stock distributed from RIKEN BSI to Nagasaki University

Authentication

The cell line was not be authenticated.

Mycoplasma contamination

Mycoplasma was tested and contamination was negative.

Commonly misidentified lines
(See ICLAC register)

none

# Animals and other research organisms

Policy information about studies involving animals; ARRIVE guidelines recommended for reporting animal research, and Sex and Gender in Research

Laboratory animals

C57BL/6J male mice age 8 weeks old were obtained from CLEA Japan.Mice were housed under a 12-hour light/dark cycle at temperature 20-26°C and humidity 30-70% with sufficient ventilation.

Wild animals

This study did not involve wild animals.

Reporting on sex

We conducted recombinant α-synuclein fibril injection experiments using both male and female mice before this study. There were no significant differences of α-synuclein propagation between males and females, so in this study, we used male mice.

Field-collected samples

The study did not involve the use of field-collected samples.

Ethics oversight

The experiments were performed in accordance with the guidelines for Animal Care of Juntendo University and approved by the Juntendo University Animal Care and Use Committee (approval number: 310187).

Note that full information on the approval of the study protocol must also be provided in the manuscript.
